# Global epidemiological trends, distribution of NTDs and malaria, and disease burden projections for the next 15 years

**DOI:** 10.3389/fpubh.2026.1696185

**Published:** 2026-05-12

**Authors:** Huan Huang, Yang Gao, Huajing Zhu, Yameng Xu, Minli Li, Fanjin Meng, Xinyan Chen, Gengting Du, Weibo Ma, Jiaojiao Qian, Yuanyuan Li, Weilong Tan

**Affiliations:** 1 Department of Epidemiology, School of Public Health, Nanjing Medical University, Nanjing, China; 2 Nanjing Bioengineering (Gene) Technology Centre for Medicines, Nanjing, China; 3 Center for Disease Control and Prevention in Xishuangbanna Dai Autonomous Prefecture, Jinghong, China; 4 Jinling Hospital, Nanjing, China

**Keywords:** environmental risks, global disease burden, malaria, neglected tropical diseases, VAR model

## Abstract

**Background:**

This study investigates global and regional trends in the burden of malaria and neglected tropical diseases (NTDs) from 1990 to 2021, focusing on their changing patterns and influencing factors. Using data from the Global Burden of Disease study 2021 (GBD study 2021), we analyzed the incidence, prevalence, mortality, disability-adjusted life years (DALYs), and age-standardized rates (ASRs) of malaria and NTDs at both regional and global levels. A vector autoregression (VAR) model was employed to explore the effects of the Socio-Demographic Index (SDI), environmental risks, and behavioral risks and to predict the disease burden over the next 15 years. The study highlights the geographical distribution and variations in risk factors associated with malaria and NTDs, offering public health recommendations for their elimination.

**Methods:**

This research used data from the GBD study 2021 to examine annual incidence rates (age-standardized incidence rate, ASIR), mortality rates (age-standardized death rate, ASDR), and Disability-Adjusted Life Years (DALYs) for malaria and NTDs by region, age, and sex. Joinpoint software was applied to analyze time trends from 1990 to 2021. A VAR model incorporating factors such as the SDI, unsafe water sources, sanitation, and handwashing practices was employed to identify significant risk factors influencing disease burden and to project future trends.

**Results:**

From 1990 to 2021, the global burden of malaria decreased, especially in Sub-Saharan Africa, while the incidence of NTDs increased until 2016 and then decreased. Age-standardized DALYs and ASDR for NTDs showed a steady decline, reflecting the impact of effective interventions. The VAR model showed that the SDI influenced both malaria and NTD burdens, especially in low-SDI regions, with environmental sanitation and unsafe behavioral factors significantly impacting malaria.

**Conclusion:**

Strengthening environmental sanitation and international cooperation can reduce the burden of malaria and NTDs. Future public health efforts should focus on enhancing infrastructure, particularly in low-SDI regions, to combat challenges posed by climate change and to support the ongoing elimination of malaria and NTDs.

## Background

Malaria is a globally pervasive infectious disease transmitted to humans by specific types of mosquitoes, causing over 600,000 deaths annually, with approximately 90% of these fatalities occurring in children under 5 years of age (1). Nearly half of the world’s population is at risk of malaria infection. Similarly, neglected tropical diseases (NTDs) comprise a group of parasitic, bacterial, and viral infections that disproportionately affect impoverished and resource-limited communities and received limited attention until the early 21st century (2, 3). More than 1 billion people worldwide are affected by at least one NTD, thereby leading to approximately 534,000 deaths and 57 million disability-adjusted life years (DALYs) lost annually (4). The World Health Organization (WHO) estimated that more than 1.6 billion people require at least one intervention for NTDs every year (5).

NTDs, along with malaria, HIV, and tuberculosis, are often collectively referred to as “infectious diseases of poverty” (IDoP) (6). These diseases are predominantly concentrated in rural regions of Sub-Saharan Africa, Asia, and Latin America, where overlapping geographic distributions frequently lead to high rates of co-infection (7–9). Consequently, integrated control strategies for NTDs and malaria are regarded as effective public health interventions. According to the GBD study 2021, malaria and NTDs are classified within the same category, and their global burden is comprehensively assessed under this framework.

Since 2000, significant progress has been made in global malaria control, primarily supported by increased funding for insecticide-treated nets (ITNs), indoor residual spraying, chemoprevention, diagnostic testing, and other effective interventions (6). However, climate change, global trade, vector re-distribution, and global warming are enhancing the potential transmission capacity of vector-borne diseases, thereby increasing the risk of malaria, dengue, schistosomiasis, and other infections (10). In addition, factors such as inadequate public health infrastructure, low education levels, and poor sanitation have undergone major shifts over recent decades, potentially impacting the global burden and trends of NTDs and malaria.

Although numerous studies have assessed the burden and trends of either NTDs or malaria, these studies are typically limited to a single disease, country, or region. Consequently, a comprehensive investigation of the global burden and trends of both NTDs and malaria is urgently needed, especially considering variations across different geographic regions, age groups, and sexes. In-depth analyses of these disease trends can support more effective allocation of health resources and the development of targeted intervention strategies.

This study utilizes data from the Global Burden of Disease (GBD) study 2021 to analyze the incidence, mortality, and DALYs associated with prevalent NTDs and malaria at global, regional, and national levels. Joinpoint regression analysis was employed to explore temporal and age-specific trends from 1990 to 2021. In addition, a vector autoregression (VAR) model, incorporating the Socio-Demographic Index (SDI)—a composite measure of per capita income, educational attainment, and total fertility rate in a country—was used to project disease burden trends over the next 15 years (2022 to 2036). This approach allows assessment of how disease burden varies with socioeconomic development and other factors and provides a scientific basis for future integrated control strategies.

## Methods

### Overview

#### Data source and framework

This study utilized data from the GBD study 2021, which provides incidence and mortality estimates for 369 diseases and injuries across 204 countries and territories from 1990 to 2021. The GBD study employs multiple methods to assess fatal and non-fatal health outcomes: Non-fatal incidence rates are estimated using DisMod-MR 2.1, a Bayesian regression analysis tool, while fatal outcomes are estimated using the Cause of Death Ensemble Model (CODEm). Input data for this study can be accessed through the Global Health Data Exchange (GHDx) query tool. This research primarily focuses on data for 18 NTDs and malaria from the GBD study, which together account for over 80% of total global NTD-related DALYs (see Supplementary Table 1). The variables extracted include annual incidence cases, age-standardized incidence rate (ASIR), age-standardized mortality rate (ASMR), and age-standardized DALYs, each with a 95% uncertainty interval (UI).

#### Data processing and metric calculations

To quantify trends in disease burden, the ASIR was used to calculate the estimated annual percentage change (EAPC). The ASIR was standardized according to the following formula, enabling cross-sectional comparisons across different age structures and providing a foundation for disease trend analysis:


$$\begin{array}{l} \mathrm{Age}\;\;S\;\tan\;\text{dardized}\;\;\text{Rate}\;(\mathrm{per}\;100,000\;\text{population})=\\ \frac{\sum_{i=1}^{A}a_{i}w_{i}}{\sum_{i=1}^{A}w_{i}}\times 100,000 \end{array}$$

*a_i_* (where i denotes the i-th age subgroup) is the age-specific rate for each age group, and *w_i_* is the weight (proportion) of the i-th age subgroup in the chosen reference population, calculated as the ratio of the number of people in that age group to the total standard population. The age-standardized rate (ASR) trend can serve as a useful proxy for changes in disease patterns within a population and provide insights into changes in disease risk factors.

#### Trend analysis

A Joinpoint regression model (Joinpoint software version 5.2.0) was employed to analyze trends in disease incidence and mortality rates. By identifying inflection points within the time series, we segmented the trends into multiple segments and used the annual percent change (APC) and average annual percent change (AAPC) to characterize these trend segments. Statistical significance for the APC and AAPC was set at a *p*-value of < 0.05.

#### Prediction of malaria and NTD burdens and endogenous variables

To forecast the age-standardized DALY rate for malaria and NTDs from 2022 to 2036, we applied a VAR model to capture dynamic relationships among multiple variables through the lagged values of each variable within the system (11). The VAR(*p*) model is specified as a system of K interrelated equations: $Y_{t}=C+\beta_{1}Y_{t-1}+\beta_{2}Y_{t-2}+\ldots +\beta_{p}Y_{t-p}+\mathrm{\varepsilon t},\;\mathrm{\varepsilon t}\sim N(0,\Sigma )$ .

In the VAR model, ***Y***_***t***_ **= (y_**1t**_, y_**2t**_, …, y_**kt**_)** is a K × 1 vector of endogenous variables at time t; **C** is a K × 1 vector of intercept terms; and*β_1_*,*β_2_*, …,*β_p_* are K × K matrices of autoregressive coefficients. The element (i, m) of the matrix *βᵢ* captures the effect of a one-unit change in the m-th variable at lag i on the current value of the i-th variable. ϵ_t_ = (ε_1t_, ε_2t_, …, ε_kt_), is a K × 1 vector of white noise innovations, satisfying ϵ_t_ ~ N(0, *Σ*), where Σ is a K × K positive definite covariance matrix.

The VAR model for NTDs and malaria can be expressed as follows:

1. The age-standardized DALY rate for malaria:

$$\begin{array}{l} Y_{t}=(\mathrm{DAL}Y_{t},\mathrm{SD}I_{t},\text{WAS}H_{t},\text{UnsafeSe}x_{t}),\\ \mathrm{DAL}Y_{t}=c_{1}+\beta_{11}^{\left(1\right)}\mathrm{DAL}Y_{t-1}+\beta_{12}^{\left(1\right)}\mathrm{SD}I_{t-1}+\beta_{13}^{\left(1\right)}\text{UnsafeSe}x_{t-1}+\\ \beta_{14}^{\left(1\right)}\text{WAS}H_{t-1}+\beta_{11}^{\left(2\right)}\mathrm{DAL}Y_{t-2}+\beta_{12}^{\left(2\right)}\mathrm{SD}I_{t-2}+\beta_{13}^{\left(2\right)}\text{UnsafeSe}x_{t-2}+\\ \beta_{14}^{\left(2\right)}\text{WAS}H_{t-2}+\varepsilon_{1t},\\ \mathrm{SD}I_{t}=c_{2}+\beta_{21}^{\left(1\right)}\mathrm{DAL}Y_{t-1}+\beta_{22}^{\left(1\right)}\mathrm{SD}I_{t-1}+\beta_{23}^{\left(1\right)}\text{UnsafeSe}x_{t-1}+\\ \beta_{24}^{\left(1\right)}\text{WAS}H_{t-1}+\beta_{21}^{\left(2\right)}\mathrm{DAL}Y_{t-2}+\beta_{22}^{\left(2\right)}\mathrm{SD}I_{t-2}+\\ \beta_{23}^{\left(2\right)}\text{UnsafeSe}x_{t-2}+\beta_{24}^{\left(2\right)}\text{WAS}H_{t-2}+\varepsilon_{2t},\\ \text{UnsafeSe}x_{t}=c_{3}+\beta_{31}^{\left(1\right)}\mathrm{DAL}Y_{t-1}+\beta_{32}^{\left(1\right)}\mathrm{SD}I_{t-1}+\\ \beta_{33}^{\left(1\right)}\text{UnsafeSe}x_{t-1}+\beta_{34}^{\left(1\right)}\text{WAS}H_{t-1}+\beta_{31}^{\left(2\right)}\mathrm{DAL}Y_{t-2}+\\ \beta_{32}^{\left(2\right)}\mathrm{SD}I_{t-2}+\beta_{33}^{\left(2\right)}\text{UnsafeSe}x_{t-2}+\beta_{34}^{\left(2\right)}\text{WAS}H_{t-2}+\varepsilon_{3t},\\ \text{WAS}H_{t}=c_{4}+\beta_{41}^{\left(1\right)}\mathrm{DAL}Y_{t-1}+\beta_{42}^{\left(1\right)}\mathrm{SD}I_{t-1}+\\ \beta_{43}^{\left(1\right)}\text{UnsafeSe}x_{t-1}+\beta_{44}^{\left(1\right)}\text{WAS}H_{t-1}+\beta_{41}^{\left(2\right)}\mathrm{DAL}Y_{t-2}+\\ \beta_{42}^{\left(2\right)}\mathrm{SD}I_{t-2}+\beta_{43}^{\left(2\right)}\text{UnsafeSe}x_{t-2}+\beta_{44}^{\left(2\right)}\text{WAS}H_{t-2}+\varepsilon_{4t}. \end{array}$$

(2) The age-standardized DALY rate for NTDs:


$$\begin{array}{l} Z_{t}=(\mathrm{DAL}Y_{t},\mathrm{SD}I_{t}),\\ \mathrm{DAL}Y_{t}=c_{1}+\gamma_{11}^{\left(1\right)}\mathrm{DAL}Y_{t-1}+\gamma_{12}^{\left(1\right)}\mathrm{SD}I_{t-1}+\\ \gamma_{11}^{\left(2\right)}\mathrm{DAL}Y_{t-2}+\gamma_{12}^{\left(2\right)}\mathrm{SD}I_{t-2}+v_{1t}\\ \mathrm{SD}I_{t}=c_{2}+\gamma_{21}^{\left(1\right)}\mathrm{DAL}Y_{t-1}+\gamma_{22}^{\left(1\right)}\mathrm{SD}I_{t-1}+\\ \gamma_{21}^{\left(2\right)}\mathrm{DAL}Y_{t-2}+\gamma_{22}^{\left(2\right)}\mathrm{SD}I_{t-2}+v_{2t} \end{array}$$

We employed the R programming language to construct a VAR model. The variable selection process was rigorously conducted in two stages to ensure methodological robustness. An initial set of candidate predictors included 15 secondary risk factors from the GBD 2021 dataset, including the SDI, unsafe sex, unsafe water, sanitation, and handwashing, air pollution, child and maternal malnutrition, dietary risks, drug use, high alcohol use, high body mass index, high fasting plasma glucose, low physical activity, non-optimal temperature, and tobacco use.

First, to address multicollinearity, we performed a correlation analysis and excluded variables with pairwise correlation coefficients exceeding a threshold of 0.8 (Supplementary Figures S1–S4). Subsequently, to avoid the inappropriate removal of theoretically important determinants, we consulted the established scientific literature on malaria and NTDs. This step ensured the retention of variables with empirically supported causal relationships with disease burden, even in cases where they were statistically correlated with other predictors.

**Figure 1:**
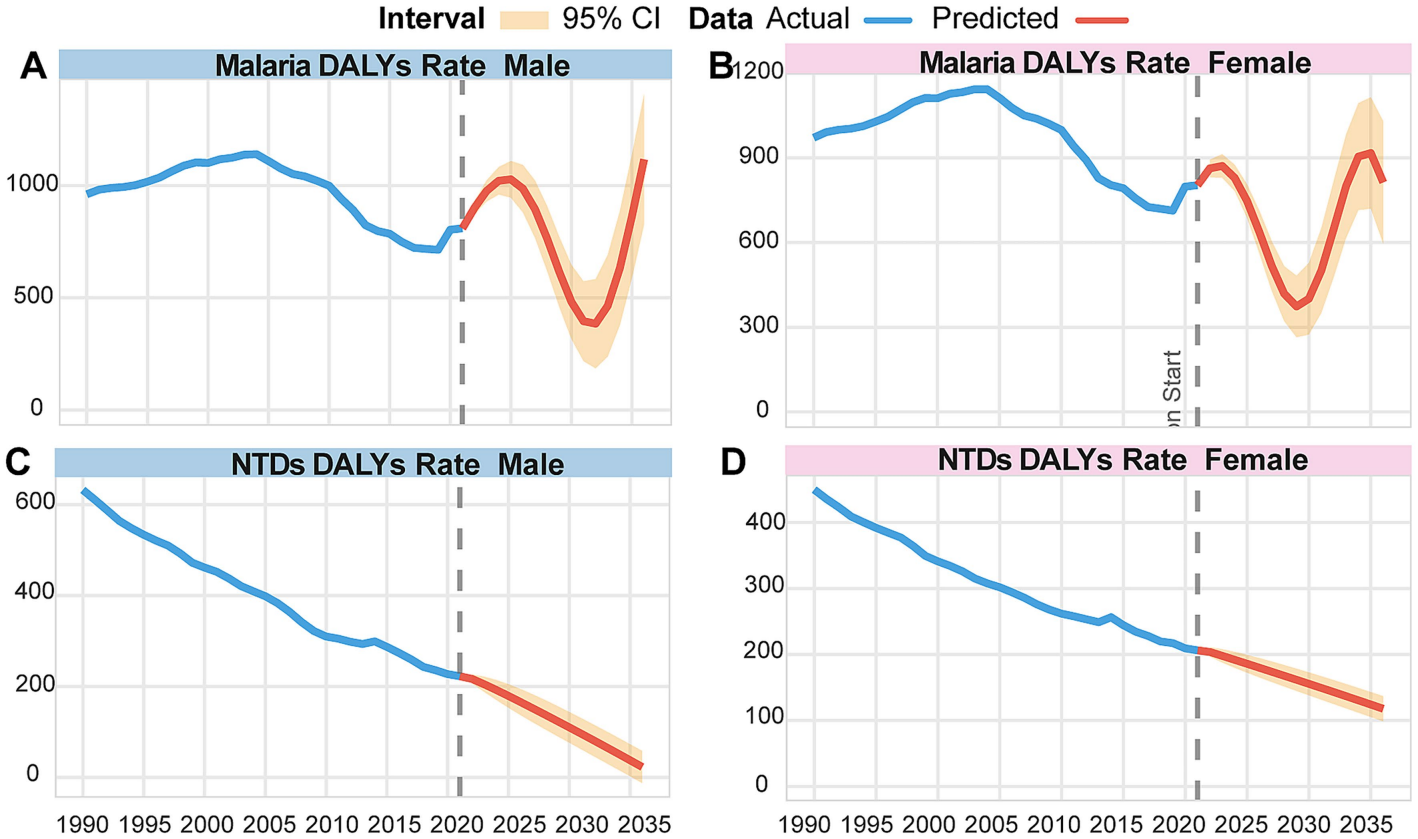
Age-standardized DALY rates of NTDs and malaria, and observed versus predicted values for each endogenous variable (with 95% confidence intervals, 1990–2036). All endogenous variables were jointly forecast; DALY rates are shown for emphasis. Malaria: **(A)** Male, **(B)** Female; Neglected tropical diseases (NTDs): **(C)** Male, **(D)** Female.

Following this two-stage selection protocol, the finalized variable sets were standardized. The model for malaria ultimately incorporated the age-standardized DALY rate and three additional predictors, while the model for NTDs included the age-standardized DALY rate and two other variables. Prior to model estimation, the stationarity of each included time series was confirmed using the augmented Dickey–Fuller (ADF) unit root test. Finally, model predictive performance was rigorously evaluated using a rolling window forecasting approach. The estimated VAR (2) models were used to generate recursive forecasts for all endogenous variables in each system for the period 2022–2036. The predicted trajectories of DALY rates (by sex) and the other endogenous variables are jointly presented in Figure 1.

#### Lag order selection and model specification

The optimal lag order for the VAR model was determined using a multi-criteria decision framework. Although information criteria (AIC, BIC, HQ, and FPE) computed for lags 1–10 initially suggested six lags, the limited sample size (*n* = 32) rendered a VAR (6) specification (requiring 100 parameters) prone to overfitting. Therefore, we focused on evaluating more parsimonious specifications (lags 1–4) based on residual diagnostics (Portmanteau test for autocorrelation), model stability (inverse roots of the characteristic polynomial), and out-of-sample forecast performance (RMSE and MAPE). The VAR (2) model was selected as it met the key diagnostic criteria while maintaining parsimony. Johansen cointegration tests confirmed the presence of three cointegrating relationships among the four variables.

#### Model diagnostics and limitations

Comprehensive diagnostic checks were performed on the selected VAR (2) model. The residuals showed no significant autocorrelation or heteroskedasticity but did exhibit non-normality—a common feature of epidemiological data that does not invalidate point estimates. Recursive CUSUM tests indicated structural stability, and Granger causality tests revealed bidirectional causality among variables, supporting the VAR framework. We acknowledge several limitations, primarily the small sample size, which constrains asymptotic properties and necessitates a parsimonious specification. The presence of cointegration suggests that a vector error correction model could provide additional insights into long-run relationships; however, the VAR (2) model remains suitable for medium-term forecasting. Despite non-negligible forecast uncertainty (MAPE = 19.89%), sensitivity analyses with alternative lag orders and variable sets confirmed the robustness of our qualitative findings, particularly the multi-factorial determinants of malaria burden. Overall, our approach represents a rigorous application of multivariate time series methods to epidemiological forecasting under realistic data constraints.

#### Statistical analysis and software tools

All data analyses and visualizations were conducted using R version 4.4.1 and Joinpoint version 5.2.0. Both the ASIR and the DALY rate are presented as the number of cases per 100,000 population, with statistical significance set at a *p*-value of < 0.05. Since this study used publicly available secondary data containing no personally identifiable information, ethical approval was not required.

## Results

### Global, regional, and National Burden Trends of NTDs and malaria from the GBD study 2021

In 2021, the global number of malaria cases was 170 million, accounting for 15% of the total disease burden. Compared to 1990, malaria incidence increased by 9%, rising from 160 million cases in 1990 to 170 million cases in 2021. However, the age-standardized incidence rate (ASIR) decreased by 16%, from 2,797 cases per 100,000 population in 1990 to 2,336 cases per 100,000 population in 2021. The age-standardized DALY rate also decreased by 16.5%, from 58 million years in 1990 to 55 million years in 2021. In contrast, the global number of NTD cases in 2021 was 940 million. Compared to 1990, the incidence of NTDs decreased by 46.6%, and the age-standardized incidence rate declined by 61.1%, from 31,461.7 cases per 100,000 population in 1990 to 12,250.7 cases per 100,000 population in 2021 (Table 1).

**Table 1:** Disease burden and disability-adjusted life year (DALYs) loss for NTDs and malaria in 2021, and the percentage change in age-standardized rates (ASRs) per 100,000 population by global burden of disease (GBD) region from 1990 to 2021.

Disease	Location	Prevalence (95% UI)			DALYs (95% UI)		
		Count (95% UI)	ASR per 105 (95% UI)	Percentage change in ASRs from 1990 to 2021	Count (95% UI)	ASR per (105 5% UI)	Percentage change in ASRs from 1990 to 2021
Malaria	Global	173885570.1 (157990967.4 to 194458400.3)	2336.8 (2122.9 to 2612.2)	−16.5 (−18.5 to −15.1)	55174060.7 (21761299.6 to 108337905.8)	806 (318.9 to 1570.2)	−16.5 (−36.5 to −17.3)
	High-income Asia Pacific	10469.2 (5898.2 to 17478.9)	6.6 (3.7 to 11)	−50.3 (−59 to −41.4)	62 (51.1 to 76.5)	0 (0 to 0)	−97.6 (−97.6 to −97.5)
	High-income North America	0 (0 to 0)	0 (0 to 0)	-	0 (0 to 0)	0 (0 to 0)	-
	Western Europe	0 (0 to 0)	0 (0 to 0)	-	0 (0 to 0)	0 (0 to 0)	−100 (−100 to −100)
	Australasia	0 (0 to 0)	0 (0 to 0)	-	0 (0 to 0)	0 (0 to 0)	-
	Andean Latin America	159180.1 (57045.8 to 205797.9)	241 (86.3 to 311.5)	−81.9 (−90.4 to −87.8)	2,154 (798.3 to 7,401)	3.2 (1.2 to 11.1)	−98.7 (−97.9 to −98.3)
	Tropical Latin America	271433.7 (199857.5 to 361,295)	126.6 (92.7 to 168.1)	−73.9 (−77.9 to −70)	13026.3 (3188.4 to 33877.5)	6 (1.5 to 15.6)	−96.5 (−97.7 to −95.4)
	Central Latin America	507785.3 (392898.2 to 649040.1)	203.5 (156.7 to 260.6)	−56.9 (−65 to −47.2)	31985.9 (7411.7 to 75722.6)	13 (3 to 30.8)	−69.5 (−86.4 to −61.1)
	Southern Latin America	0 (0 to 0)	0 (0 to 0)	−100 (−100 to −100)	0 (0 to 0)	0 (0 to 0)	−100 (−100 to −100)
	Caribbean	65303.6 (37392.5 to 129234.1)	144.5 (82.3 to 287.2)	−22.8 (−44.6 to −3.5)	47765.2 (7055.6 to 148307.2)	103.7 (15.4 to 321.9)	−29.4 (−71.8 to −6.7)
	Central Europe	0 (0 to 0)	0 (0 to 0)	-	0 (0 to 0)	0 (0 to 0)	-
	Eastern Europe	0 (0 to 0)	0 (0 to 0)	-	0 (0 to 0)	0 (0 to 0)	-
	Central Asia	0 (0 to 0)	0 (0 to 0)	−100 (−100 to −100)	0 (0 to 0)	0 (0 to 0)	−100 (−100 to −100)
	North Africa and Middle East	2867996.5 (1,962,985 to 4292673.6)	450.3 (308.2 to 674.1)	−30.4 (−35.1 to −31.2)	786625.4 (237427.9 to 1,697,098)	125.5 (37.8 to 272.1)	−35.8 (−49 to −40.9)
	South Asia	5970785.2 (4920191.9 to 7,803,469)	321.3 (264.8 to 419.9)	−77.9 (−73.7 to −82.7)	1820079.6 (126366.2 to 6010614.1)	104.1 (6.9 to 344.8)	−82.6 (−97 to −82.3)
	Southeast Asia	1704659.7 (1211199.3 to 1951376.1)	248.3 (176 to 284.1)	−73 (−76.5 to −76.9)	110524.3 (34865.9 to 363321.6)	16.1 (5.2 to 52.1)	−90.9 (−89.5 to −92.7)
	East Asia	31,885 (28396.6 to 35605.7)	2.4 (2.2 to 2.7)	−96.2 (−94 to −96.5)	224.6 (163.7 to 308.6)	0 (0 to 0)	−99.7 (−95.3 to −100)
	Oceania	830376.4 (745698.5 to 921833.2)	5622.3 (5,047 to 6,243)	−56 (−10.7 to −82.5)	146,292 (66771.8 to 266,018)	1047.5 (474.8 to 1931)	−60.1 (−18.3 to −75.9)
	Western Sub-Saharan Africa	95093771.8 (78815387.7 to 110970343.2)	17,793 (14749.2 to 20773.1)	−46.8 (−51 to −44.7)	33222938.6 (13351431.2 to 63411625.4)	5668.4 (2216.2 to 11127.5)	−31.1 (−48.2 to −21.6)
	Eastern Sub-Saharan Africa	34555455.8 (29688749.2 to 39078451.8)	7386.2 (6348.7 to 8,354)	−59.3 (−62.2 to −58.1)	11609738.7 (4520723.8 to 21788865.5)	2199.7 (830.7 to 4301.6)	−56.8 (−71.4 to −49.7)
	Central Sub-Saharan Africa	31617345.1 (23854192.2 to 42362284.6)	21328.8 (16092.3 to 28557.4)	−44 (−52 to −33.1)	7237039.5 (3416571.5 to 12922264.3)	4076.7 (1862 to 7643.6)	−50.4 (−59.8 to −41.8)

This table presents the global and regional disease burden (including incidence, mortality, and DALY loss) caused by NTDs and malaria in 2021, as well as the percentage change in age-standardized rates (ASRs) per 100,000 population by GBD region from 1990 to 2021. The data were sourced from the Global Burden of Disease (GBD) study and were extracted using the GBD Results Tool available at the following link: https://ghdx.healthdata.org/gbd-results-tool.

### Geographical and regional differences

**Figure 2:**
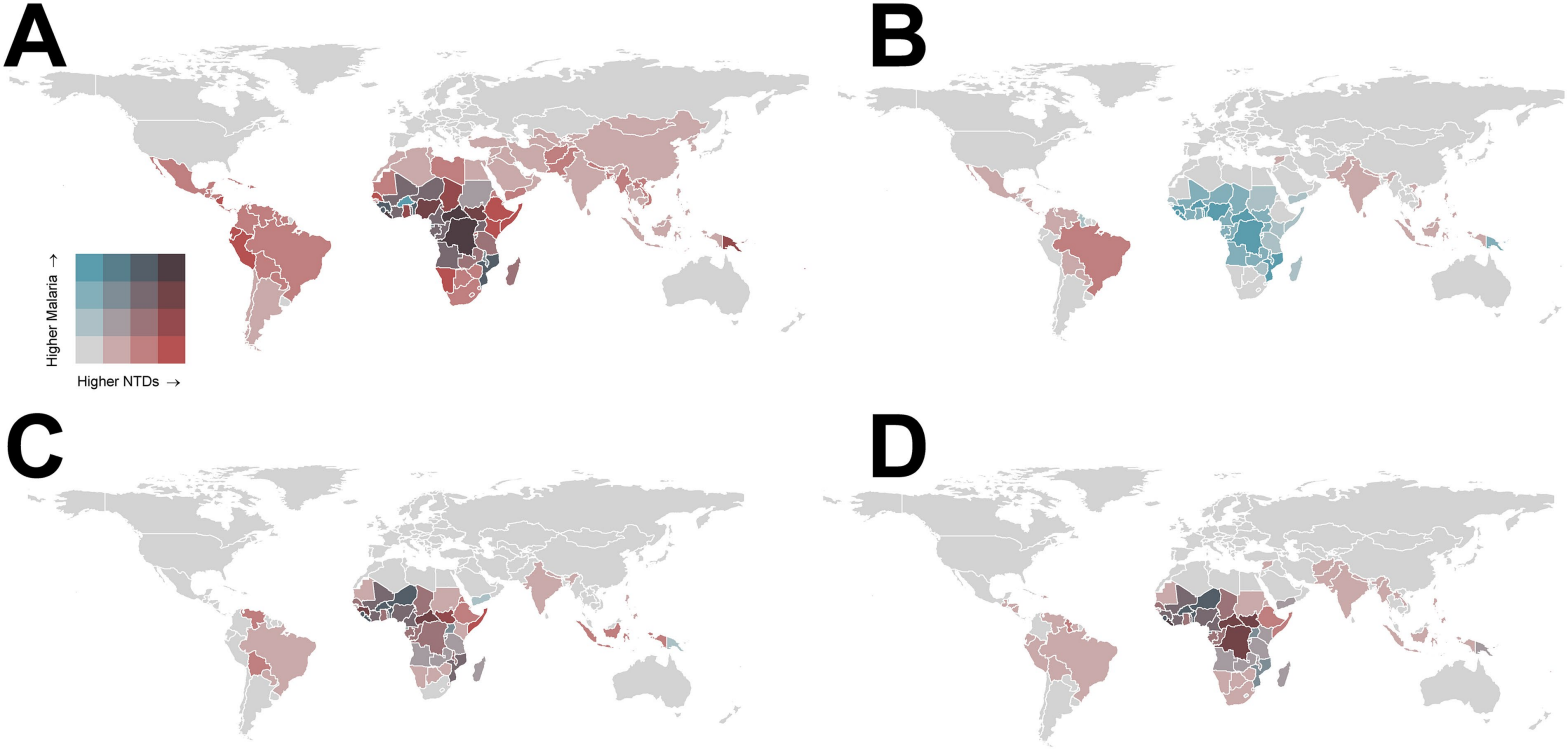
Bivariate maps showing the geographical differences in age-standardized rates (ASRs) of NTDs and malaria in 2021 (per 100,000 persons). **(A)** Age-standardized prevalence rate (ASPR); **(B)** age-standardized incidence rate (ASIR); **(C)** age-standardized death rate (ASDR); **(D)** age-standardized DALY rate. ASIR, Age-Standardized Incidence Rate; ASPR, Age-Standardized Prevalence Rate; ASDR, Age-Standardized Death Rate; NTDs, Neglected Tropical Diseases.

In 2021, the highest overlap in the ASPR for NTDs and malaria was observed in West and Central Sub-Saharan Africa (Figure 2A, Table 1). High ASIR areas for malaria were concentrated in Sub-Saharan Africa and Oceania, while regions with higher NTD ASIRs were concentrated in tropical Latin America, Andean Latin America, South Asia, and Southeast Asia (Figure 2B). The geographic patterns for the age-standardized death rate (ASDR) (Figure 2C) and DALYs (Figure 2D) were similar, with the highest overlaps observed in Sub-Saharan Africa and Oceania. The ASDR and DALYs for both NTDs and malaria were higher in Sub-Saharan Africa, while NTDs showed a higher ASDR in South Asia, Southeast Asia, and tropical Latin America. In contrast, the malaria ASDR was higher in Southern Sub-Saharan Africa and Oceania (Figures 2C,D).

### Temporal trends of NTDs and malaria

**Figure 3:**
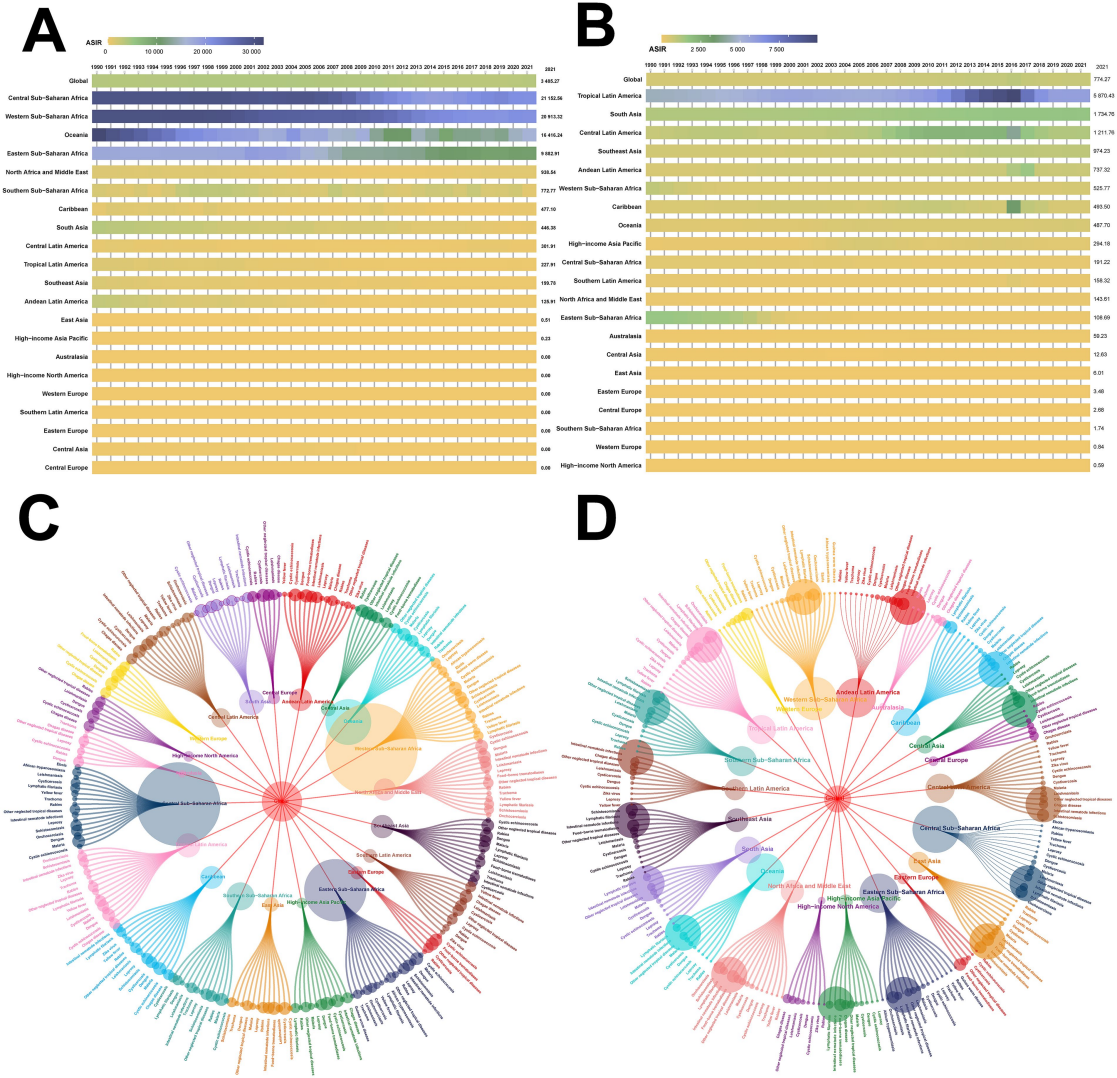
Trends and disease burden composition of the ASIR for NTDs and malaria globally and across 21 GBD regions from 1990 to 2021. **(A)** Trends in the ASIR of malaria globally and across 21 GBD regions from 1990 to 2021; **(B)** trends in the ASIR of NTDs globally and across 21 GBD regions from 1990 to 2021; **(C)** composition of DALYs for NTDs and malaria across 21 GBD regions in 2021; **(D)** composition of prevalence for NTDs and malaria across 21 GBD regions in 2021.

From 1990 to 2021, the malaria ASIR remained consistently high in Central, West, and East Sub-Saharan Africa, showing an overall downward trend, while Oceania showed a fluctuating decline (Figure 3A). NTDs exhibited a high ASIR in tropical Latin America, South Asia, and Central Latin America, with notable fluctuations from 2012 to 2016, peaking in 2016 before gradually declining thereafter (Figure 3B).

**Figure 4:**
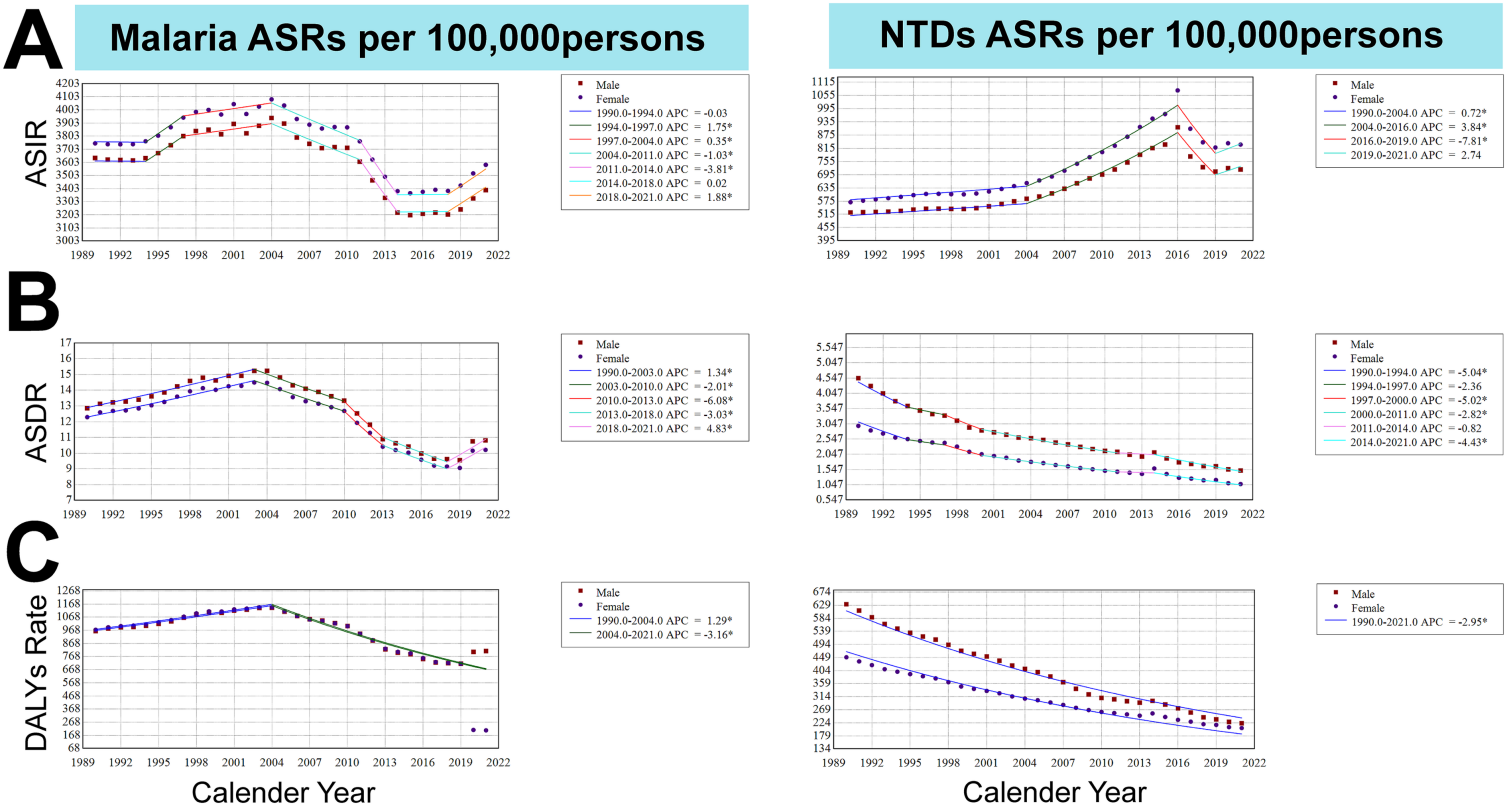
Trends in age-standardized rates (ASRs) of malaria and NTDs globally from 1990 to 2021. **(A)** ASIR; **(B)** ASDR; **(C)** Age-standardized DALY rate.

Joinpoint regression analysis revealed a global decline in the malaria ASIR, ASDR, and age-standardized DALY rate for both sexes from 1990 to 2021. Segmental trends showed that the malaria ASIR was higher among female individuals, decreasing during 2004–2011 and 2011–2014 but rising during 1994–1997, 1997–2004, and 2018–2021. The malaria ASDR was higher among male individuals, with declines during 2003–2010, 2010–2013, and 2013–2018 and increases during 1990–2003 and 2018–2021. The age-standardized DALY rate for malaria showed minimal sex differences, rising from 1990 to2004 (APC = 1.29) and declining from 2004 to 2021 (APC = −3.16) (Figure 4).

For NTDs, the ASIR showed an overall increase from 1990 to 2021 and was higher among female individuals, with segmented increases during 1990–2004 and 2004–2016 and a decline during 2016–2019. The ASDR and age-standardized DALY rate for NTDs trended downward, with male individuals consistently higher than female individuals (Figure 4).

### Disease composition and the correlation of NTD and malaria ASIRs with the SDI

Among the 21 regions in the GBD study, malaria accounted for the largest proportion of DALYs for NTDs and malaria combined. NTDs, dengue, and lymphatic filariasis represented the highest DALY burdens, especially in South Asia, Southeast Asia, and Sub-Saharan Africa. Food-borne trematodiases also contributed significantly to the burden in East Asia (Figure 3C).

For disease composition, intestinal nematode infections dominate, particularly in South Asia, West and East Sub-Saharan Africa, and Latin America, with East Asia also showing a high prevalence of intestinal nematode infections, foodborne trematodiases, and schistosomiasis. Malaria remains highly concentrated in West, East, and Central Sub-Saharan Africa, areas that also show a high burden of schistosomiasis (Figure 3D).

**Figure 5:**
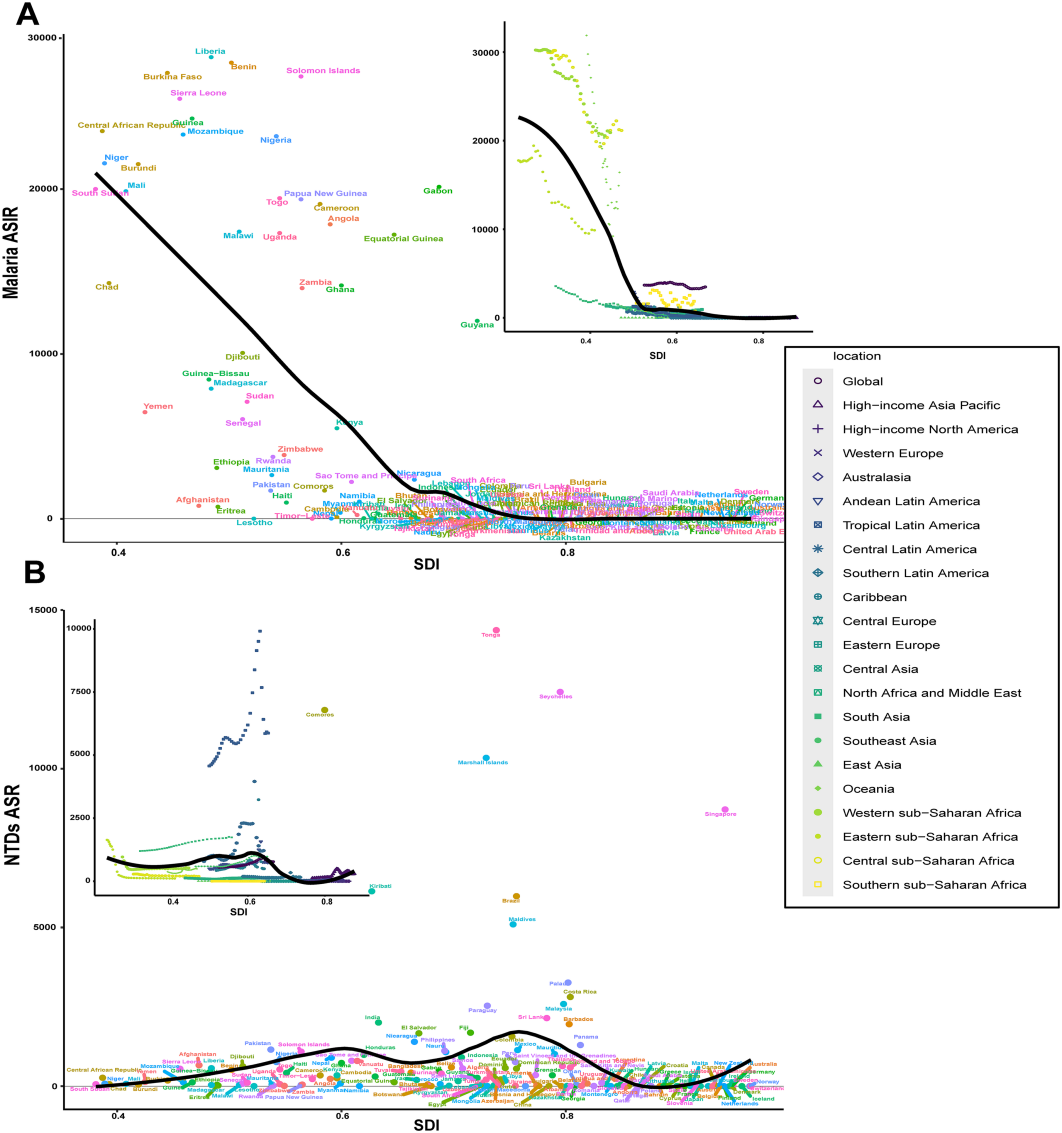
ASIRs of NTDs and malaria across 21 GBD regions in 204 countries and territories, in relation to the SDI, at the global, regional, and country levels in 2021 **(A)** Malaria; **(B)** NTDs.

The regional ASIR of NTDs from 1990 to 2021 closely followed the expected levels based on the SDI across the globe and in many regions. However, the Tropical and Central Latin America regions and South Asia exhibited significant deviations from this trend, displaying much higher incidence rates. Southeast Asia also demonstrated an increasing trend (Figure 5B). In malaria observations, a negative correlation was identified between the ASIR and the SDI, with a turning point of approximately 0.5 (Figure 5A). Trends in the SDI and ASIR at both national and regional levels displayed more consistent patterns (Figures 5A,B).

### Global ASRs of NTDs and malaria by sex prediction

The VAR model predicted that the age-standardized DALY rate for malaria will continue to rise, peaking in 2025, followed by a gradual decline. A trough is expected in 2033, after which a slow resurgence will occur. By 2036, the rate is projected to reach 521.73 per 100,000 for male individuals and 519.62 per 100,000 for female individuals.

In contrast, for NTDs, the age-standardized DALY rates for both male and female individuals are predicted to decrease steadily from 2022 to 2036 (Figure 1). Regarding the other variables incorporated into the VAR model, the global SDI showed an upward trend, while the rate of unsafe water, sanitation, and hygiene WASH exhibited a downward trend. For the burden attributable to unsafe sex, female DALYs are projected to decline, whereas male DALYs are projected to rise initially before declining, with a peak expected around 2029 (Figure 1).

## Discussion

### Epidemiological and geographical trends

This study delineates the evolving global burden of malaria and NTDs from 1990 to 2021 and projects the burden over the following 15 years. Our analysis reveals divergent trajectories: While the burden of malaria has shown a general decline worldwide, the ASIR of NTDs increased until approximately 2016 before beginning to decrease. Notably, for NTDs, both the age-standardized DALY rate and ASDR demonstrated a continuous decline over the entire period, suggesting that public interventions have been effective in reducing disease severity and mortality even as incidence fluctuated.

Over the past 30 years, significant progress has been made in the global fight against malaria. Between 2000 and 2023, an estimated 2.2 billion malaria cases and 12.7 million malaria deaths were averted worldwide, with 1.7 billion cases and 12 million deaths prevented in the WHO African Region alone. During this period, the mortality rate dropped by 50% (5). Children under 5 years of age remain the most vulnerable group to malaria. The reduction in the malaria burden can largely be attributed to the use of insecticide-treated nets (ITNs) and increased coverage of preventive antimalarial drugs for pregnant women and children. Data show that the proportion of pregnant women and children using insecticide-treated nets in Sub-Saharan Africa increased from 26% in 2010 to 61% in 2018, which has effectively reduced malaria prevalence and its DALY burden. Despite overall improvements, progress in high-burden areas remains limited, while climate change, the migration of vector species, and the increase in global travel and trade have expanded the geographic distribution of the disease. The emergence of drug resistance continues to be a major challenge in malaria control (12).

In contrast, the global burden profile of NTDs is characterized by a rise in the ASIR until 2016, driven notably by regions such as tropical Latin America. Schistosomiasis and intestinal nematode infections constitute the majority (>85%) of this burden. The observed peak around 2016, followed by a decline in incidence, aligns temporally with the intensification of global control efforts following the 2012 WHO roadmap. The concurrent, steady decrease in age-standardized DALYs and the ASDR indicates the significant impact of large-scale interventions, including mass drug administration (MDA), international cooperation, drug donations, and national control strategies (13). For instance, while intestinal nematode infections are highly prevalent, their disability weight is relatively low, and widespread deworming campaigns have effectively reduced their severe outcomes. Despite this success, NTDs are also affected by the expanding geographic range of their vectors and pathogens due to climatic and environmental changes (14).

A critical comparative analysis revealed distinct demographic patterns in disease burden. Malaria exerts its highest toll on children under 5 years of age, while NTDs predominantly affect children under 10. This difference underscores the age-specific windows of vulnerability related to exposure and immunity. However, both diseases are profoundly linked to socioeconomic determinants, functioning as archetypal poverty-related conditions. The SDI is an important factor, yet transmission dynamics are amplified by interconnected drivers such as climate change, unplanned urbanization, population displacement, and inadequate water, sanitation, and hygiene infrastructure. These factors collectively create ecologies favorable for vectors and facilitate transmission through both direct contact and environmental contamination.

This epidemiological synthesis concludes that, while targeted interventions for NTDs and malaria have been distinct and disease-specific, their shared determinants suggest that integrated, cross-cutting strategies—particularly those focusing on maternal and child health, environment management, and poverty alleviation—could yield synergistic benefits in reducing the overall burden of both disease groups. The following section explores the multivariate modeling approach that quantifies these complex interrelationships.

### Modeling and forecasting interpretation (VAR model)

To disentangle the complex, multi-driver dynamics underpinning the burden of NTDs and malaria, we employed a VAR model. This approach is predicated on the recognition that infectious disease transmission operates within a system of interrelated variables, including socio-demographic shifts, environmental factors, and public health interventions, which continuously influence one another over time. Unlike univariate time series models, which analyze a single series in isolation, as a mainstay of multivariate time series analysis, the VAR framework allows for the joint analysis of these multiple endogenous variables. It is uniquely capable of quantifying the dynamic feedback and temporal interdependencies among them, such as how improvements in the SDI may subsequently affect exposure risks, which, in turn, collectively determine disease burden trajectories.

Sukono et al. applied a vector autoregressive model with exogenous variables to forecast the COVID-19 pandemic, incorporating vaccination rates as exogenous variables alongside endogenous variables such as confirmed cases and recovery rates. Their analysis identified the VARX (7, 1) specification as the optimal model, which outperformed univariate time series models significantly (15). This study corroborated the VAR model’s capacity to effectively disentangle the interactions between exogenous interventions and endogenous variables, clarifying the lagged effects of exogenous factors on disease burden. Consequently, the VAR model provides a more realistic theoretical framework for understanding how multiple determinants collectively shape epidemiological trends. Compared to univariate models, it offers methodological advantages in forecasting complex systems, where critical inter-variable interactions may otherwise be overlooked.

Guided by this framework, we constructed separate VAR models to forecast age-standardized DALY rates for malaria and NTDs. The optimal model specification was determined through a systematic comparison of alternative lag structures (*p* = 1–4), evaluated based on residual diagnostics, stability, and out-of-sample predictive accuracy (Supplementary Table S3). A lag order of 2 [VAR (2)] was selected as it best balanced statistical adequacy with model parsimony. The final set of variables for each model was informed by both statistical correlations and established epidemiological evidence. For the malaria model, the selected predictors included the target DALY rate, the SDI, the burden attributable to unsafe sex, and the burden linked to WASH. For the NTD model, variables related to water, sanitation, and handwashing were considered based on established associations (16, 17) but were ultimately not retained in the final parsimonious specification, as their exclusion did not significantly degrade model fit, suggesting that the SDI may capture their relevant variance in this context. A rolling window forecasting exercise was conducted for out-of-sample validation, yielding a low RMSE and indicating robust predictive performance (18). This aligns with comparative studies suggesting that, while multivariate models may pose greater estimation challenges, their overall forecasting accuracy for complex systems often surpasses that of univariate autoregressive models (19–21).

The model results revealed several meaningful and distinct relationships between variables. Within the malaria model, the burden attributable to unsafe sex emerged as a significant predictor. This finding hints at a potential associative pathway beyond traditional vector-borne transmission dynamics. One plausible explanatory mechanism could involve immunological susceptibility or health system factors related to co-infection. For instance, the notable geographical overlap between malaria and HIV, a disease primarily transmitted through unsafe sex, is well documented, and co-infection with these diseases is known to exacerbate adverse outcomes and likely increase the composite DALY burden (22). This association warrants further investigation to clarify the direction and mechanisms of this relationship. Meanwhile, the distinct role of WASH is particularly instructive, which was an indispensable contributor in the malaria model but less critical in the NTD model. This divergence suggests that, for malaria, these specific environmental exposures represent direct risks not fully captured by the broader SDI, a finding supported by studies linking poor WASH conditions to higher malaria risk among children in low-SDI settings (23). In contrast, the burden of NTDs appears to be more strongly governed by systemic health capacities and large-scale intervention programs encompassed by the SDI.

The forecasting outputs projected that the global age-standardized DALY rate for malaria would peak around 2025 before declining, with a tentative gradual resurgence observed after 2033. It is important to underscore that long-term forecasts are inherently accompanied by widening confidence intervals and that trends beyond 2030 are subject to greater uncertainty. This nuanced projection, a potential plateauing or reversal of gains, resonates with warnings from the World Malaria Report regarding stalled progress and the risk of resurgence in some regions. For NTDs, the model forecast a steady decline in burden, aligning with the positive trajectory supported by intensified global control efforts, including the notable achievement that 50 countries had eliminated at least one NTD by 2023, marking significant progress toward the 2030 roadmap goals (24).

We acknowledge several limitations that contextualize our findings. The principal constraint lies in the temporal scope of the data. Although the dataset spans 32 years, from 1990 to 2021, the number of observed time points remains limited for fitting a multi-parameter VAR model, which may affect the stability of parameter estimates and long-term forecast precision. Furthermore, regarding variable selection, although the SDI is a powerful composite index, the model does not incorporate more proximate climate variables (e.g., temperature and rainfall), whose effects on malaria dynamics are well established (25, 26), nor does it include more granular intervention coverage data. Future research could significantly extend this study by integrating climate projection scenarios or policy dummy variables into a VAR framework for scenario-based forecasting or by employing methods such as Bayesian VAR to enhance robustness when working with limited time series data. Such advancements would further strengthen the utility of systems-oriented modeling in planning resilient disease control strategies.

## Conclusion

This study showed contrasting global epidemiological trends for malaria and neglected tropical diseases (NTDs) from 1990 to 2021. The overall burden of malaria decreased globally, while NTD incidence rose until 2016 and then declined, with sustained reductions in age-standardized DALY and mortality rates for both diseases. The burden remains concentrated in Sub-Saharan Africa, South Asia, and tropical Latin America. The SDI is a key driver of both diseases; malaria is additionally affected by unsafe water, sanitation, and hygiene conditions and risky sexual behaviors, whereas NTDs are more strongly determined by socioeconomic development. VAR projections indicated that malaria DALYs would peak around 2025 and then decline, while NTD DALYs will decrease steadily through 2036. Strengthening water/sanitation infrastructure, improving socioeconomic conditions, and implementing integrated control strategies in low-SDI regions are critical for sustaining reductions in disease burden and advancing global elimination goals.

## Data Availability

The original contributions presented in the study are included in the article/Supplementary material, further inquiries can be directed to the corresponding authors.
