# Supplementary material for: Global epidemiological trends, distribution of NTDs and malaria, and disease burden projections for the next 15 years
Source: Front Public Health. 2026 May 12;14:1696185. doi: 10.3389/fpubh.2026.1696185 (PMC13201495; doi:10.3389/fpubh.2026.1696185)
Supplement: Supplementary file 1 [file Supplementary_file_1.docx]

| **Diseases** | **Location** | **Prevalence(95%UI)** | | | **DALYs(95%UI)** | | |
| --- | --- | --- | --- | --- | --- | --- | --- |
|  |  | **Numbers(95%UI)** | **ASRs per 100000(95%UI)** | **Percentage Change in ASRs from1990 to 2021** | **Numbers(95%UI)** | **ASRs per 100000(95%UI)** | **Percentage Change in ASRs from1990 to 2021** |
| **Chagas disease** |  |  |  |  |  |  |  |
|  | Global | 6297840·7 (5422631·7 to 7206339·4) | 75·6 (65·1 to 86·5) | -47·9 (-48 to -48·6) | 238589·3 (207472·1 to 271774·8) | 2·8 (2·4 to 3·2) | -99·8 (-99·8 to -99·7) |
|  | High-income Asia Pacific | 5587·9 (4835·8 to 6440·7) | 2·5 (2·1 to 2·9) | 192·9 (189·7 to 190·3) | 61·4 (39·8 to 94) | 0 (0 to 0) | -100 (-100 to -100) |
|  | High-income North America | 246107·7 (205725·6 to 289035·8) | 55·9 (46·4 to 65·7) | -1·9 (-2·5 to -2·8) | 2644·8 (1713·4 to 3902·7) | 0·5 (0·3 to 0·8) | 4652·3 (5285·4 to 4276·2) |
|  | Western Europe | 43595·2 (37958·3 to 49431·5) | 8·4 (7·2 to 9·5) | 120·1 (118·7 to 117·7) | 466·7 (306 to 713·3) | 0·1 (0 to 0·1) | 1283·1 (1263·6 to 1276·9) |
|  | Australasia | 1698·3 (1433 to 1994·8) | 4·6 (3·9 to 5·4) | -58·2 (-59·8 to -56·7) | 18·3 (11·9 to 27·4) | 0 (0 to 0·1) | -100 (-100 to -100) |
|  | Andean Latin America | 810181·1 (720545 to 901577) | 1224·5 (1089·3 to 1362·4) | -63·2 (-62·3 to -64·8) | 17723·9 (12996·3 to 23932·3) | 28·9 (21·2 to 39·1) | -100 (-100 to -100) |
|  | Tropical Latin America | 2090637·1 (1799849·7 to 2384371·9) | 830·8 (715·1 to 945·9) | -48·2 (-47·7 to -49·4) | 153604·9 (136074·6 to 172363·9) | 59·3 (52·5 to 66·5) | -100 (-100 to -100) |
|  | Central Latin America | 2074658·1 (1738420·8 to 2416133·9) | 791 (663·3 to 920·2) | -47·8 (-48·1 to -49·2) | 44670 (34789·7 to 57449·7) | 17·6 (13·7 to 22·6) | -100 (-100 to -100) |
|  | Southern Latin America | 1023447·6 (890660·9 to 1170380·9) | 1346·1 (1176·1 to 1538·4) | -65·9 (-66·1 to -65·1) | 19376 (14832·6 to 25478·4) | 23·5 (17·9 to 31) | -100 (-100 to -100) |
|  | Caribbean | 1916·6 (1646·5 to 2211·7) | 3·7 (3·2 to 4·3) | -44·8 (-45·4 to -44·8) | 23·2 (15·5 to 33·8) | 0 (0 to 0·1) | 945·2 (1078·9 to 899) |
|  | Central Europe | 10·9 (8·5 to 13·9) | 0 (0 to 0) | - | 0·1 (0·1 to 0·2) | 0 (0 to 0) | - |
|  | Eastern Europe | 0 (0 to 0) | 0 (0 to 0) | - | 0 (0 to 0) | 0 (0 to 0) | - |
|  | Central Asia | 0 (0 to 0) | 0 (0 to 0) | - | 0 (0 to 0) | 0 (0 to 0) | - |
|  | North Africa and Middle East | 0 (0 to 0) | 0 (0 to 0) | - | 0 (0 to 0) | 0 (0 to 0) | - |
|  | South Asia | 0 (0 to 0) | 0 (0 to 0) | - | 0 (0 to 0) | 0 (0 to 0) | - |
|  | Southeast Asia | 0 (0 to 0) | 0 (0 to 0) | - | 0 (0 to 0) | 0 (0 to 0) | - |
|  | East Asia | 0 (0 to 0) | 0 (0 to 0) | - | 0 (0 to 0) | 0 (0 to 0) | - |
|  | Oceania | 0 (0 to 0) | 0 (0 to 0) | - | 0 (0 to 0) | 0 (0 to 0) | - |
|  | Western Sub-Saharan Africa | 0 (0 to 0) | 0 (0 to 0) | - | 0 (0 to 0) | 0 (0 to 0) | - |
|  | Eastern Sub-Saharan Africa | 0 (0 to 0) | 0 (0 to 0) | - | 0 (0 to 0) | 0 (0 to 0) | - |
|  | Central Sub-Saharan Africa | 0 (0 to 0) | 0 (0 to 0) | - | 0 (0 to 0) | 0 (0 to 0) | - |
|  | Southern Sub-Saharan Africa | 0 (0 to 0) | 0 (0 to 0) | - | 0 (0 to 0) | 0 (0 to 0) | - |
| **Leishmaniasis** |  |  |  |  |  |  |  |
|  | Global | 6209395·7 (5825066·4 to 6694050·9) | 77 (71·9 to 83) | 22·3 (35·8 to 8·3) | 781187·6 (468168·2 to 1636591·7) | 10·3 (6 to 22) | -99 (-99·9 to -98·2) |
|  | High-income Asia Pacific | 0 (0 to 0) | 0 (0 to 0) | - | 0 (0 to 0) | 0 (0 to 0) | - |
|  | High-income North America | 412 (254·4 to 692) | 0·1 (0·1 to 0·2) | 10·7 (198·7 to -32) | 27·6 (14·2 to 50) | 0 (0 to 0) | 595998·4 (1692908·8 to 319547·6) |
|  | Western Europe | 4138·7 (3224 to 5622·1) | 0·8 (0·6 to 1) | -23·8 (-0·4 to -41·6) | 1645·6 (593 to 8296·4) | 0·5 (0·2 to 2·7) | -97·4 (-92·9 to -99·4) |
|  | Australasia | 0 (0 to 0) | 0 (0 to 0) | - | 0 (0 to 0) | 0 (0 to 0) | - |
|  | Andean Latin America | 357493·9 (317878·2 to 403132·3) | 548·7 (487·3 to 619·8) | -0·6 (25·2 to -20·4) | 23161·7 (15488·1 to 32892·5) | 35·5 (23·8 to 50·6) | -43·2 (-37·7 to -56) |
|  | Tropical Latin America | 736074·7 (667661·7 to 817839·6) | 297·4 (269·8 to 330·7) | -1·7 (15·1 to -15·3) | 114002·5 (36297·4 to 294025·1) | 52·5 (14·8 to 143·5) | -98·6 (-96·4 to -99·1) |
|  | Central Latin America | 552350·1 (494120·9 to 640115·6) | 215·9 (193·2 to 249·7) | -10·5 (13·3 to -29) | 36034·5 (23656·3 to 52082·9) | 14·1 (9·3 to 20·5) | 36·4 (45·9 to 19) |
|  | Southern Latin America | 9481·1 (7454·8 to 13116·5) | 12·8 (10·3 to 17·5) | -1·3 (100·1 to -38·9) | 656·7 (368 to 1168·9) | 0·9 (0·5 to 1·8) | 1067 (3002·5 to 337·3) |
|  | Caribbean | 26183 (21334·1 to 33100·8) | 52·9 (43·2 to 66·2) | -4·3 (43·9 to -32·2) | 1661·4 (1079 to 2444·3) | 3·4 (2·2 to 4·9) | 54·1 (-55·6 to 275·9) |
|  | Central Europe | 1021·5 (840·7 to 1295·2) | 0·7 (0·5 to 0·8) | -50·6 (-43·5 to -57·1) | 385·6 (48·1 to 2980·3) | 0·4 (0 to 3·8) | -94·2 (134 to -96·6) |
|  | Eastern Europe | 0 (0 to 0) | 0 (0 to 0) | - | 0 (0 to 0) | 0 (0 to 0) | - |
|  | Central Asia | 103350·2 (86278·1 to 124641·1) | 111·9 (93·5 to 135·5) | -48·9 (-44·5 to -53·1) | 8870·8 (4428·4 to 27278·6) | 9·4 (4·8 to 28·3) | -100 (-100 to -100) |
|  | North Africa and Middle East | 3831680·5 (3531870 to 4202149·4) | 642·9 (592·1 to 712·9) | -0·4 (23·1 to -19·3) | 272525·6 (170483·8 to 492504·4) | 45·2 (28·4 to 79·8) | -90·2 (-82·8 to -94·5) |
|  | South Asia | 342973·7 (292951·5 to 406609·2) | 18·9 (16·2 to 22·6) | -20·7 (6·7 to -36·1) | 106311·7 (15610·7 to 478610·3) | 5·9 (0·9 to 26·1) | -100 (-100 to -100) |
|  | Southeast Asia | 14510·3 (12370 to 16710·4) | 2·1 (1·8 to 2·4) | 91·1 (226 to 24·2) | 1028 (626·1 to 1517·7) | 0·1 (0·1 to 0·2) | -96·1 (-91·1 to -97·8) |
|  | East Asia | 20540·3 (10591·8 to 44225·6) | 1·1 (0·6 to 2·2) | -56 (-45·4 to -61·6) | 9822 (761·6 to 48835·6) | 0·8 (0 to 4·4) | 908·8 (15017·3 to 409·6) |
|  | Oceania | 0 (0 to 0) | 0 (0 to 0) | - | 0 (0 to 0) | 0 (0 to 0) | - |
|  | Western Sub-Saharan Africa | 154722·3 (137889·6 to 173270·8) | 41·9 (36·6 to 48·4) | 17·5 (65·6 to -12·3) | 29889·1 (19044·3 to 44056·8) | 5·5 (3·7 to 7·8) | -85·2 (-98·2 to -18·2) |
|  | Eastern Sub-Saharan Africa | 36553 (31755·6 to 42207·8) | 11 (9·2 to 13·6) | -57·3 (-46 to -63·1) | 127772·8 (76095·5 to 194043·5) | 23 (13·9 to 34·6) | -98·4 (-98·6 to -97·6) |
|  | Central Sub-Saharan Africa | 17412·8 (13745·8 to 22288·8) | 16·2 (12·3 to 22·3) | -40·7 (-9 to -55·6) | 47360·2 (24257·4 to 77017·5) | 26·8 (14 to 43·1) | -100 (-100 to -100) |
|  | Southern Sub-Saharan Africa | 497·5 (390·4 to 640·2) | 0·7 (0·5 to 0·9) | -9·9 (63 to -40·2) | 31·7 (19·4 to 46·8) | 0 (0 to 0·1) | 221·5 (421·2 to 113·7) |
| **Schistosomiasis** |  |  |  |  |  |  |  |
|  | Global | 151376744·5 (109062891·2 to 198666395·8) | 1914·3 (1378·9 to 2510·9) | -22·6 (-31·5 to -15·1) | 1746333·3 (1038122·1 to 2984204·2) | 21·9 (12·9 to 37·3) | -39·4 (-50·6 to -30·8) |
|  | High-income Asia Pacific | 0 (0 to 0) | 0 (0 to 0) | - | 0 (0 to 0) | 0 (0 to 0) | - |
|  | High-income North America | 0 (0 to 0) | 0 (0 to 0) | - | 0 (0 to 0) | 0 (0 to 0) | - |
|  | Western Europe | 0 (0 to 0) | 0 (0 to 0) | - | 0 (0 to 0) | 0 (0 to 0) | - |
|  | Australasia | 0 (0 to 0) | 0 (0 to 0) | - | 0 (0 to 0) | 0 (0 to 0) | - |
|  | Andean Latin America | 0 (0 to 0) | 0 (0 to 0) | - | 0 (0 to 0) | 0 (0 to 0) | - |
|  | Tropical Latin America | 5841735·2 (4317716·1 to 7647498·6) | 2395·4 (1751·9 to 3105·6) | -14 (-15·6 to -13·8) | 55910·6 (33102·9 to 95820·7) | 22·6 (13·2 to 39·5) | -100 (-100 to -100) |
|  | Central Latin America | 744418·8 (509565·3 to 1057606·7) | 280·8 (192 to 399·1) | -18·3 (-29·9 to -7·5) | 6405·1 (3428·9 to 12420·5) | 2·4 (1·3 to 4·7) | -100 (-100 to -100) |
|  | Southern Latin America | 0 (0 to 0) | 0 (0 to 0) | - | 0 (0 to 0) | 0 (0 to 0) | - |
|  | Caribbean | 1246510·4 (829969·3 to 1802980·4) | 2567·4 (1709·8 to 3683·9) | 16·3 (14·7 to 17·3) | 9921·4 (4738·8 to 21091·5) | 20·3 (9·6 to 43·3) | -100 (-100 to -100) |
|  | Central Europe | 0 (0 to 0) | 0 (0 to 0) | - | 0 (0 to 0) | 0 (0 to 0) | - |
|  | Eastern Europe | 0 (0 to 0) | 0 (0 to 0) | - | 0 (0 to 0) | 0 (0 to 0) | - |
|  | Central Asia | 0 (0 to 0) | 0 (0 to 0) | - | 0 (0 to 0) | 0 (0 to 0) | - |
|  | North Africa and Middle East | 7289692·4 (5158092 to 10390474·4) | 1127·9 (805·1 to 1597·3) | -83·3 (-87·2 to -77·9) | 84796·2 (49989·3 to 151196·7) | 13·9 (8·5 to 24·2) | -96·8 (-97·4 to -96·2) |
|  | South Asia | 0 (0 to 0) | 0 (0 to 0) | - | 0 (0 to 0) | 0 (0 to 0) | - |
|  | Southeast Asia | 1052615 (717565·8 to 1797568·2) | 142·8 (97·3 to 244·1) | -59·9 (-61·5 to -64·3) | 17805 (12662·3 to 27701·7) | 2·4 (1·7 to 3·8) | 1306·2 (1150·8 to 1361) |
|  | East Asia | 11459581·1 (8953887 to 15054894·9) | 733·9 (559·3 to 955·9) | -40 (-42·5 to -43·6) | 86748·8 (43666·1 to 167202·4) | 5·3 (2·6 to 10·6) | -67·9 (-78·2 to -57·9) |
|  | Oceania | 0 (0 to 0) | 0 (0 to 0) | - | 0 (0 to 0) | 0 (0 to 0) | - |
|  | Western Sub-Saharan Africa | 61534853·9 (44035019·9 to 81338005·3) | 13433·6 (9651·8 to 17812·1) | -42·1 (-46·7 to -38·6) | 710978·9 (413882·5 to 1212784·3) | 171·1 (106·5 to 289·2) | -54·9 (-54 to -57·3) |
|  | Eastern Sub-Saharan Africa | 46949044·3 (33903446 to 61048520·1) | 11617·6 (8558 to 15093·1) | -42·4 (-47·2 to -38·4) | 552262·6 (332712·5 to 948844) | 157·6 (103·6 to 261·7) | -64·8 (-70·3 to -57·3) |
|  | Central Sub-Saharan Africa | 10268752·6 (7147340·9 to 13878619·3) | 7947·5 (5702·6 to 11151·9) | -58·4 (-61·3 to -52·6) | 165861·4 (113450·1 to 255589·9) | 161·9 (116·5 to 234·2) | -100 (-100 to -100) |
|  | Southern Sub-Saharan Africa | 4989540·7 (3609448·3 to 6699969·6) | 5951 (4379·2 to 7964·8) | -22·9 (-25 to -20·5) | 55643·3 (32529·7 to 98937·1) | 68·8 (41·6 to 120·9) | -84·6 (-84·4 to -84·3) |
| **African trypanosomiasis** |  |  |  |  |  |  |  |
|  | Global | 2367·9 (1130·4 to 4391·7) | 0 (0 to 0·1) | -97·4 (-97·3 to -97·5) | 61962·5 (22232·7 to 176263·8) | 0·8 (0·3 to 2·3) | -100 (-100 to -100) |
|  | High-income Asia Pacific | 0 (0 to 0) | 0 (0 to 0) | - | 0 (0 to 0) | 0 (0 to 0) | - |
|  | High-income North America | 0 (0 to 0) | 0 (0 to 0) | - | 0 (0 to 0) | 0 (0 to 0) | - |
|  | Western Europe | 0 (0 to 0) | 0 (0 to 0) | - | 0 (0 to 0) | 0 (0 to 0) | - |
|  | Australasia | 0 (0 to 0) | 0 (0 to 0) | - | 0 (0 to 0) | 0 (0 to 0) | - |
|  | Andean Latin America | 0 (0 to 0) | 0 (0 to 0) | - | 0 (0 to 0) | 0 (0 to 0) | - |
|  | Tropical Latin America | 0 (0 to 0) | 0 (0 to 0) | - | 0 (0 to 0) | 0 (0 to 0) | - |
|  | Central Latin America | 0 (0 to 0) | 0 (0 to 0) | - | 0 (0 to 0) | 0 (0 to 0) | - |
|  | Southern Latin America | 0 (0 to 0) | 0 (0 to 0) | - | 0 (0 to 0) | 0 (0 to 0) | - |
|  | Caribbean | 0 (0 to 0) | 0 (0 to 0) | - | 0 (0 to 0) | 0 (0 to 0) | - |
|  | Central Europe | 0 (0 to 0) | 0 (0 to 0) | - | 0 (0 to 0) | 0 (0 to 0) | - |
|  | Eastern Europe | 0 (0 to 0) | 0 (0 to 0) | - | 0 (0 to 0) | 0 (0 to 0) | - |
|  | Central Asia | 0 (0 to 0) | 0 (0 to 0) | - | 0 (0 to 0) | 0 (0 to 0) | - |
|  | North Africa and Middle East | 0 (0 to 0) | 0 (0 to 0) | - | 0 (0 to 0) | 0 (0 to 0) | - |
|  | South Asia | 0 (0 to 0) | 0 (0 to 0) | - | 0 (0 to 0) | 0 (0 to 0) | - |
|  | Southeast Asia | 0 (0 to 0) | 0 (0 to 0) | - | 0 (0 to 0) | 0 (0 to 0) | - |
|  | East Asia | 0 (0 to 0) | 0 (0 to 0) | - | 0 (0 to 0) | 0 (0 to 0) | - |
|  | Oceania | 0 (0 to 0) | 0 (0 to 0) | - | 0 (0 to 0) | 0 (0 to 0) | - |
|  | Western Sub-Saharan Africa | 935·8 (356·3 to 1914) | 0·2 (0·1 to 0·5) | -93·7 (-93·7 to -93·7) | 19122·5 (6970·2 to 38822·6) | 4·1 (1·5 to 8·4) | -100 (-100 to -100) |
|  | Eastern Sub-Saharan Africa | 404·7 (242 to 654·8) | 0·1 (0·1 to 0·2) | -98·8 (-98·5 to -99) | 24148·4 (3281·6 to 120468·8) | 5·8 (0·8 to 28·9) | -100 (-100 to -100) |
|  | Central Sub-Saharan Africa | 1027·4 (524·1 to 1805·5) | 0·9 (0·5 to 1·6) | -99 (-98·9 to -99·1) | 18691·5 (9275 to 32271·2) | 15 (7·4 to 25·9) | -99·9 (-100 to -99·9) |
|  | Southern Sub-Saharan Africa | 0 (0 to 0) | 0 (0 to 0) | - | 0 (0 to 0) | 0 (0 to 0) | - |
| **Cystic echinococcosis** |  |  |  |  |  |  |  |
|  | Global | 148520·9 (119837·7 to 183224·2) | 1·8 (1·5 to 2·3) | -13·8 (-22 to -7·3) | 105071·6 (78966·6 to 133308·9) | 1·3 (1 to 1·7) | -53·2 (-60 to -48·3) |
|  | High-income Asia Pacific | 357·7 (263·8 to 497·5) | 0·2 (0·1 to 0·2) | 5·1 (5·8 to 3·8) | 139·9 (90·3 to 205·6) | 0·1 (0 to 0·1) | 619·1 (533·8 to 674·4) |
|  | High-income North America | 840·4 (616·2 to 1122) | 0·2 (0·1 to 0·3) | 1491·8 (1688·8 to 1332) | 403·7 (271·2 to 557·3) | 0·1 (0·1 to 0·1) | 37·1 (31·4 to 37·4) |
|  | Western Europe | 2827·4 (2344·4 to 3355·4) | 0·5 (0·5 to 0·6) | -25·5 (-22·9 to -27·4) | 1356·7 (971·5 to 1841·4) | 0·2 (0·2 to 0·3) | -90·6 (-91·9 to -89·6) |
|  | Australasia | 90·5 (69·8 to 115·8) | 0·2 (0·2 to 0·3) | -65 (-65·8 to -65·1) | 48·4 (33·6 to 64) | 0·1 (0·1 to 0·2) | 37·6 (33·9 to 38) |
|  | Andean Latin America | 350 (279·9 to 434·1) | 0·5 (0·4 to 0·7) | 22·3 (22·5 to 24·7) | 195·8 (135·9 to 269·8) | 0·3 (0·2 to 0·4) | 14·4 (32·1 to 9·3) |
|  | Tropical Latin America | 879·9 (682·8 to 1111·3) | 0·4 (0·3 to 0·4) | 557·2 (569·5 to 540·6) | 535·5 (362·6 to 729·6) | 0·2 (0·1 to 0·3) | 61·3 (78·1 to 57·3) |
|  | Central Latin America | 1254·3 (939·7 to 1680·9) | 0·5 (0·4 to 0·6) | -61·9 (-63·4 to -60·7) | 486·6 (323·3 to 668·1) | 0·2 (0·1 to 0·3) | -84·5 (-85·6 to -83·8) |
|  | Southern Latin America | 6421·2 (5389·8 to 7567·4) | 8·8 (7·5 to 10·4) | -98·6 (-98·8 to -98·3) | 2215·5 (1508·9 to 3091·5) | 3 (2 to 4·2) | -98·1 (-98·2 to -98·1) |
|  | Caribbean | 40·8 (31·6 to 51·4) | 0·1 (0·1 to 0·1) | 370·5 (378·5 to 358·6) | 26·2 (17·6 to 36·6) | 0·1 (0 to 0·1) | 45·6 (80 to 21·9) |
|  | Central Europe | 3411·8 (2852·4 to 4061·7) | 2·5 (2·1 to 3) | 5·3 (7·1 to 4·9) | 1458·6 (1030·9 to 1974·1) | 1 (0·7 to 1·4) | -4·1 (-1·8 to -6·5) |
|  | Eastern Europe | 8824·9 (7037·2 to 10846) | 3·5 (2·8 to 4·2) | 30·1 (32 to 29·1) | 3692·8 (2563·2 to 5130·4) | 1·4 (1 to 1·9) | -91·7 (-93·1 to -90·3) |
|  | Central Asia | 5119·7 (4108·1 to 6268·2) | 5·3 (4·3 to 6·4) | -29·8 (-36 to -24·7) | 2105·8 (1411·2 to 2903·3) | 2·2 (1·5 to 3) | -62 (-61·6 to -61·7) |
|  | North Africa and Middle East | 22626·8 (18383·8 to 27833·8) | 3·6 (3 to 4·4) | 88 (91·9 to 75·2) | 17319·4 (13164·8 to 21974·3) | 3 (2·3 to 3·7) | -89·1 (-90·8 to -87·7) |
|  | South Asia | 48465·3 (38357·7 to 60349·7) | 2·6 (2·1 to 3·3) | -85·4 (-86 to -85) | 34005·9 (25804·2 to 43189·8) | 1·9 (1·5 to 2·4) | -44·2 (-49·2 to -40·7) |
|  | Southeast Asia | 1502·1 (1163·3 to 1945·7) | 0·2 (0·2 to 0·3) | -6·1 (-5·3 to -6·4) | 3321·1 (2163·1 to 4592·2) | 0·5 (0·3 to 0·7) | -86·9 (-86·7 to -86·5) |
|  | East Asia | 25203·3 (18892·6 to 32671·8) | 1·5 (1·1 to 2) | 187·7 (209·8 to 169·9) | 10558 (7002·9 to 14416·8) | 0·6 (0·4 to 0·8) | 113·3 (68 to 155·2) |
|  | Oceania | 3·8 (2·4 to 5·9) | 0 (0 to 0) | 3·4 (7·5 to 5·7) | 26·2 (14·8 to 42) | 0·2 (0·1 to 0·3) | -4·7 (12·6 to -11·6) |
|  | Western Sub-Saharan Africa | 1724·5 (1283·4 to 2286·3) | 0·3 (0·3 to 0·4) | -30·9 (-23·8 to -44·4) | 7342·9 (3870·5 to 12374·2) | 1·3 (0·7 to 1·9) | -81 (-83·2 to -80·2) |
|  | Eastern Sub-Saharan Africa | 17418·3 (13340·6 to 22392·8) | 4 (3·2 to 5) | -92·1 (-92·7 to -91·7) | 17337·5 (12042·2 to 23167·2) | 4·1 (3 to 5·4) | -86 (-85·2 to -86·9) |
|  | Central Sub-Saharan Africa | 962·6 (730·6 to 1258·5) | 0·7 (0·6 to 0·9) | 304·2 (302·6 to 293) | 1868 (1187 to 2641·3) | 1·5 (1 to 2) | -97·7 (-97·9 to -97·6) |
|  | Southern Sub-Saharan Africa | 195·6 (151·8 to 256·6) | 0·2 (0·2 to 0·3) | -10·6 (-10·5 to -10·9) | 627·2 (365·3 to 916) | 0·8 (0·5 to 1·2) | -96·2 (-95·5 to -96·5) |
| **Cysticercosis** |  |  |  |  |  |  |  |
|  | Global | 4357190 (3150315·7 to 5716430·4) | 51·3 (37·2 to 67·3) | -25·1 (-24 to -24) | 1235939 (787769·5 to 1808363·2) | 14·6 (9·3 to 21·3) | -39·9 (-48·8 to -31·1) |
|  | High-income Asia Pacific | 53393·9 (37526·8 to 73170·4) | 16·1 (10·9 to 22·9) | -2·6 (-1·9 to 1·8) | 10898·1 (4130·4 to 22405·7) | 3·4 (1·2 to 7·2) | -28·1 (-44·9 to -15·3) |
|  | High-income North America | 455786·6 (325720·6 to 606980·5) | 81·8 (57·5 to 108·7) | 15·5 (13·8 to 15·8) | 92482·9 (52610·2 to 150158·5) | 16·8 (9·3 to 26·9) | 125·1 (161·5 to 103·2) |
|  | Western Europe | 79323·5 (58303·2 to 105209·2) | 10·5 (7·5 to 14·2) | -3 (-2·1 to -3·4) | 15606·9 (6958·1 to 29522·1) | 2·1 (0·9 to 4) | -93·3 (-92·2 to -93·7) |
|  | Australasia | 0 (0 to 0) | 0 (0 to 0) | - | 0 (0 to 0) | 0 (0 to 0) | - |
|  | Andean Latin America | 118801·1 (89037·9 to 150771·4) | 187·4 (142·3 to 235·3) | -33 (-34·9 to -31·7) | 32595·8 (18315·5 to 53054·2) | 51·1 (29·1 to 82·3) | -41·1 (-39·8 to -42·5) |
|  | Tropical Latin America | 313300·3 (225480·5 to 409454·4) | 122 (88·7 to 159·7) | -27·8 (-27·6 to -28·5) | 87033 (55079·5 to 132764·1) | 33·9 (21·6 to 51·5) | -37·7 (-32·8 to -38·5) |
|  | Central Latin America | 562969·1 (410576·9 to 708799·2) | 216·4 (160·3 to 271·4) | -17·1 (-14·9 to -19) | 157382·2 (98522·7 to 228879·9) | 60·3 (37·9 to 87·3) | -90·6 (-91·3 to -89·1) |
|  | Southern Latin America | 65461·1 (46767·6 to 85879·9) | 80·4 (56·8 to 106·7) | -17·9 (-17·4 to -16·5) | 15617·4 (7988·9 to 26607·7) | 19·3 (9·7 to 32·8) | -32·8 (-42·4 to -26·3) |
|  | Caribbean | 54588·4 (39869 to 69310·2) | 105·3 (76·1 to 134·2) | -12·5 (-12·3 to -10·6) | 18373·1 (11745·6 to 26399·2) | 35·8 (22·8 to 51·6) | -86·9 (-88·7 to -85) |
|  | Central Europe | 147110·7 (103947·8 to 195429·8) | 81·8 (56·3 to 110·1) | -20 (-20·3 to -18·2) | 32832·1 (17876·4 to 52854) | 18·5 (10 to 29·9) | 82·7 (72 to 94·6) |
|  | Eastern Europe | 191119·5 (136087·2 to 254706·7) | 61 (43·7 to 80·6) | -33·1 (-32·1 to -33·4) | 44321·9 (24819·4 to 69985·1) | 14·3 (7·9 to 22·8) | -23·8 (-31·2 to -17·8) |
|  | Central Asia | 25166·7 (16535·1 to 36634·3) | 28·4 (19·3 to 40·8) | -35·7 (-33·6 to -34·5) | 6624·4 (3555·3 to 11164·2) | 7·4 (4 to 12·6) | -100 (-100 to -100) |
|  | North Africa and Middle East | 2621 (1655·8 to 3956·3) | 0·5 (0·3 to 0·7) | -51·9 (-50·1 to -55) | 892 (514·2 to 1469·2) | 0·2 (0·1 to 0·3) | 3484·8 (2994·7 to 3278·6) |
|  | South Asia | 755682·6 (551993·1 to 972078·6) | 51·6 (38·1 to 65·7) | -42·4 (-43·8 to -41·6) | 221269 (139957·5 to 320733·1) | 14·8 (9·5 to 21·4) | 4·6 (6·6 to 9·8) |
|  | Southeast Asia | 145176·8 (99878 to 206134·6) | 21·7 (15·3 to 30·2) | -23·4 (-24·2 to -22) | 42442·4 (24323·2 to 67083·2) | 6·2 (3·6 to 9·9) | 47·5 (49·5 to 41) |
|  | East Asia | 725883·3 (494719·5 to 1018979·9) | 35 (24·2 to 48·7) | -37·3 (-38·5 to -35·3) | 176228·2 (101464·7 to 284692·8) | 8·5 (5 to 13·7) | 2 (-6·1 to 9·5) |
|  | Oceania | 5369·1 (3392·1 to 7764·9) | 55 (37·6 to 76·7) | -33·6 (-34·6 to -31·4) | 1917·1 (1074·7 to 3110) | 19 (11·1 to 30·2) | -75·5 (-76·6 to -73) |
|  | Western Sub-Saharan Africa | 177701·3 (121380·2 to 240538·6) | 69 (50·1 to 91·2) | -25·8 (-26·4 to -24·6) | 78105·3 (50136·2 to 112896) | 26·7 (17·8 to 39) | -66·7 (-68·1 to -66) |
|  | Eastern Sub-Saharan Africa | 186229·4 (126441·3 to 256036·2) | 83·3 (61·1 to 110·3) | -35·5 (-33·9 to -34·1) | 94862·6 (64602·9 to 129427·6) | 36·4 (25·5 to 50·9) | -6 (-14·9 to -3) |
|  | Central Sub-Saharan Africa | 147074·8 (104657·2 to 189855·2) | 191·4 (144·1 to 235) | -28·7 (-29·8 to -28·8) | 58930 (37878·5 to 85786·4) | 72 (46·4 to 103·7) | -77·5 (-77·2 to -76·6) |
|  | Southern Sub-Saharan Africa | 144430·9 (101225·9 to 183711) | 208·3 (155·3 to 258·4) | -16·9 (-14·4 to -17·8) | 47524·8 (29208·3 to 67824) | 66·8 (42·7 to 94·3) | -19·4 (-20·1 to -16·1) |
| **Lymphatic filariasis** |  |  |  |  |  |  |  |
|  | Global | 56902555·8 (48666870·2 to 67914348·4) | 706 (603·7 to 841·8) | -82·6 (-82·7 to -82·6) | 1314563·4 (768842·1 to 2224976·3) | 16·5 (9·6 to 28) | -100 (-100 to -100) |
|  | High-income Asia Pacific | 4487·4 (1037·8 to 24444·3) | 2·8 (0·7 to 14·6) | 18·7 (81·3 to -14·2) | 139 (77·3 to 250·8) | 0·1 (0·1 to 0·2) | -100 (-100 to -100) |
|  | High-income North America | 0 (0 to 0) | 0 (0 to 0) | - | 0 (0 to 0) | 0 (0 to 0) | - |
|  | Western Europe | 0 (0 to 0) | 0 (0 to 0) | - | 0 (0 to 0) | 0 (0 to 0) | - |
|  | Australasia | 0 (0 to 0) | 0 (0 to 0) | - | 0 (0 to 0) | 0 (0 to 0) | - |
|  | Andean Latin America | 0 (0 to 0) | 0 (0 to 0) | - | 0 (0 to 0) | 0 (0 to 0) | - |
|  | Tropical Latin America | 18260 (15418·3 to 31865·9) | 7·9 (6·8 to 13·6) | -95·7 (-94·9 to -96·1) | 1547·6 (1069·2 to 2214·2) | 0·7 (0·5 to 1) | -97·9 (-98·2 to -97·5) |
|  | Central Latin America | 0 (0 to 0) | 0 (0 to 0) | - | 0 (0 to 0) | 0 (0 to 0) | - |
|  | Southern Latin America | 0 (0 to 0) | 0 (0 to 0) | - | 0 (0 to 0) | 0 (0 to 0) | - |
|  | Caribbean | 415467·8 (226191·9 to 805439·4) | 851·8 (465·2 to 1642·6) | -86 (-79 to -87·9) | 10123·5 (6343·7 to 16605·7) | 21·2 (13·3 to 34·9) | -80·4 (-82 to -76·9) |
|  | Central Europe | 0 (0 to 0) | 0 (0 to 0) | - | 0 (0 to 0) | 0 (0 to 0) | - |
|  | Eastern Europe | 0 (0 to 0) | 0 (0 to 0) | - | 0 (0 to 0) | 0 (0 to 0) | - |
|  | Central Asia | 0 (0 to 0) | 0 (0 to 0) | - | 0 (0 to 0) | 0 (0 to 0) | - |
|  | North Africa and Middle East | 1062017·8 (305385 to 3343336·1) | 170·4 (48·6 to 537·5) | -80 (-79·2 to -80·5) | 20401·8 (11735·1 to 36878·2) | 3·2 (1·8 to 5·8) | -100 (-100 to -100) |
|  | South Asia | 34671952 (30247621·2 to 40389153·8) | 1884·3 (1646·1 to 2191·4) | -83 (-84·1 to -81·6) | 735707·7 (428341·9 to 1239749·3) | 39·6 (23·1 to 66·4) | -100 (-100 to -100) |
|  | Southeast Asia | 7877839·9 (3890013·4 to 15733426·5) | 1089·1 (539·7 to 2167·2) | -90·2 (-91 to -88·5) | 198952·4 (114300·5 to 348217·1) | 27·8 (16 to 48·6) | -82·6 (-85·3 to -78·6) |
|  | East Asia | 0 (0 to 0) | 0 (0 to 0) | - | 0 (0 to 0) | 0 (0 to 0) | - |
|  | Oceania | 948179·2 (360722·4 to 2268683·2) | 7646·5 (2970·4 to 18140·4) | -76·8 (-82·9 to -65·6) | 19976·5 (13019·3 to 29230·8) | 163·5 (107·5 to 237·3) | -100 (-100 to -100) |
|  | Western Sub-Saharan Africa | 6926621·5 (4443188·4 to 10575998·6) | 1729·1 (1105·9 to 2662·4) | -90 (-90·2 to -89·6) | 175377 (106381·1 to 288534·9) | 41·1 (25·1 to 66·6) | -100 (-100 to -100) |
|  | Eastern Sub-Saharan Africa | 2584938·2 (1760794·6 to 3932929·2) | 702·6 (470 to 1088·8) | -94·9 (-95·2 to -94·2) | 99553·2 (58781·7 to 177185·5) | 24·6 (14·4 to 43·7) | -100 (-100 to -100) |
|  | Central Sub-Saharan Africa | 2270255·5 (1225773·2 to 4312847·1) | 1989·9 (1060·3 to 3782·6) | -86·6 (-88·1 to -83·8) | 48605·7 (27345·3 to 84824·7) | 39·1 (22·2 to 67·6) | -100 (-100 to -100) |
|  | Southern Sub-Saharan Africa | 122536·3 (42972·1 to 396129·1) | 150·9 (52·4 to 490·5) | -49·8 (-32·8 to -55·9) | 4179·1 (2422·5 to 7381·3) | 5·1 (2·9 to 8·9) | 284·2 (294·6 to 289·9) |
| **Dengue** |  |  |  |  |  |  |  |
|  | Global | 3517383·5 (928243·8 to 6430039·5) | 44·9 (11·8 to 82·1) | 56·1 (178·4 to 42·2) | 2076524·7 (1056228·4 to 3130717·7) | 27·8 (14·2 to 41·7) | -99·6 (-99·7 to -99·3) |
|  | High-income Asia Pacific | 29077·8 (5850·3 to 71641·8) | 17·6 (3·5 to 43·4) | 126·5 (231·7 to 104·2) | 4713·2 (876 to 12044·7) | 2·9 (0·5 to 7·4) | -2 (38 to 16·7) |
|  | High-income North America | 80·2 (2·4 to 402·1) | 0 (0 to 0·1) | 239·5 (-37 to 401·1) | 42·1 (15·9 to 121·1) | 0 (0 to 0) | 42151·5 (14033·9 to 60237·5) |
|  | Western Europe | 0 (0 to 0) | 0 (0 to 0) | - | 1·5 (0·5 to 2·7) | 0 (0 to 0) | 16698·7 (6157·9 to 24035·8) |
|  | Australasia | 1103 (304·2 to 2901·3) | 3·5 (1 to 9·3) | 105·8 (729·8 to 68·6) | 191·7 (49·8 to 498·3) | 0·6 (0·2 to 1·6) | 546·9 (401·3 to 939·4) |
|  | Andean Latin America | 23428·7 (9709·1 to 41697·6) | 35·5 (14·7 to 63·1) | 93·6 (601·4 to 44·4) | 4327·2 (1845·5 to 8129·2) | 6·6 (2·8 to 12·3) | 389 (1243·7 to 209·8) |
|  | Tropical Latin America | 776465 (243162·1 to 1587514·8) | 343·8 (108 to 700·3) | 29·4 (590·2 to 1·3) | 143869·6 (50995·1 to 306624·3) | 63·8 (22·4 to 136) | -100 (-100 to -100) |
|  | Central Latin America | 172010·2 (102575·6 to 247213·3) | 68 (40·5 to 97·6) | 63·7 (488·8 to 14·8) | 43074·3 (24432·8 to 67687) | 17·5 (9·9 to 27·6) | 672·2 (847·8 to 669·5) |
|  | Southern Latin America | 4765·4 (1006·9 to 11400·9) | 7·1 (1·5 to 16·9) | 54·1 (1554 to 4·2) | 819·8 (149·6 to 2211) | 1·2 (0·2 to 3·3) | -98·1 (-79·1 to -98·8) |
|  | Caribbean | 13593·9 (1664 to 38336·7) | 28·5 (3·5 to 80) | 14·9 (352·7 to 7·2) | 2751·3 (546·5 to 7216·8) | 5·8 (1·2 to 15·5) | -99·7 (-99·8 to -99·4) |
|  | Central Europe | 0 (0 to 0) | 0 (0 to 0) | - | 0 (0 to 0) | 0 (0 to 0) | - |
|  | Eastern Europe | 0 (0 to 0) | 0 (0 to 0) | - | 0 (0 to 0) | 0 (0 to 0) | - |
|  | Central Asia | 0 (0 to 0) | 0 (0 to 0) | - | 0 (0 to 0) | 0 (0 to 0) | - |
|  | North Africa and Middle East | 3192·8 (1041·6 to 11472·7) | 0·5 (0·2 to 1·8) | 63·3 (59·9 to 83·3) | 1035·1 (445·4 to 2669·4) | 0·2 (0·1 to 0·4) | 2304·4 (1703·6 to 2782·9) |
|  | South Asia | 1901418 (107573·7 to 4067252·6) | 103·2 (5·9 to 220·6) | 48·3 (705·1 to 46·1) | 931668·1 (324113·4 to 1630476·7) | 53·5 (19·5 to 91·9) | -100 (-100 to -100) |
|  | Southeast Asia | 400005·3 (281600·6 to 633454·9) | 57·8 (40·6 to 91·3) | 66·4 (508 to -5·3) | 909114·2 (589034·8 to 1241630·5) | 147 (95·3 to 201) | -96·3 (-99·7 to -91·7) |
|  | East Asia | 3636·8 (917·9 to 9167·1) | 0·3 (0·1 to 0·6) | 40·4 (260·9 to 10·6) | 1360 (568·3 to 2540·3) | 0·1 (0 to 0·2) | 1618·5 (497·3 to 3048) |
|  | Oceania | 3810·5 (1669·6 to 7578·5) | 29 (12·4 to 58·1) | 31·7 (154·5 to 2·8) | 766·8 (347·5 to 1540·5) | 6 (2·7 to 11·9) | -98·6 (-99 to -97·9) |
|  | Western Sub-Saharan Africa | 146832·4 (7218·3 to 530938·3) | 30·4 (1·5 to 111·9) | 35 (11082 to 42·1) | 24191·2 (1156·7 to 83576·2) | 4·9 (0·2 to 17) | -100 (-99·7 to -100) |
|  | Eastern Sub-Saharan Africa | 22952·7 (372·1 to 88355·2) | 5·6 (0·1 to 21·7) | -88·7 (-97·6 to -83·7) | 5940·8 (1374·6 to 17522·9) | 1·3 (0·2 to 4·1) | 1700·1 (13620·8 to 834·1) |
|  | Central Sub-Saharan Africa | 14947·3 (677 to 81023·7) | 10·9 (0·5 to 59) | 35·5 (411 to 29·7) | 2645·6 (125·9 to 14646·5) | 1·9 (0·1 to 10·3) | 380·7 (12122·1 to 46·4) |
|  | Southern Sub-Saharan Africa | 63·3 (3·3 to 428·1) | 0·1 (0 to 0·5) | 3·5 (22·2 to 3·1) | 12·3 (1·1 to 79·4) | 0 (0 to 0·1) | 3940·2 (8578·2 to 1692·4) |
|  |  |  |  |  |  |  |  |
| **Yellow Fever** | Global | 2369·7 (919·9 to 5044·5) | 0 (0 to 0·1) | -81·1 (-81·5 to -81·4) | 317791·9 (120003·9 to 686270·6) | 4·3 (1·6 to 9·2) | -100 (-100 to -100) |
|  | High-income Asia Pacific | 0 (0 to 0) | 0 (0 to 0) | - | 0 (0 to 0) | 0 (0 to 0) | - |
|  | High-income North America | 0 (0 to 0) | 0 (0 to 0) | - | 0 (0 to 0) | 0 (0 to 0) | - |
|  | Western Europe | 0 (0 to 0) | 0 (0 to 0) | - | 0 (0 to 0) | 0 (0 to 0) | - |
|  | Australasia | 0 (0 to 0) | 0 (0 to 0) | - | 0 (0 to 0) | 0 (0 to 0) | - |
|  | Andean Latin America | 29·8 (9·4 to 70·9) | 0 (0 to 0·1) | -84·8 (-86 to -84·8) | 3088·3 (778·5 to 8401·2) | 4·6 (1·2 to 12·5) | -100 (-100 to -100) |
|  | Tropical Latin America | 5·1 (2 to 12) | 0 (0 to 0) | -83·9 (-84·9 to -83·7) | 520·9 (189·8 to 1214·9) | 0·2 (0·1 to 0·6) | -83·4 (-83·8 to -83·1) |
|  | Central Latin America | 4·9 (1·6 to 11·5) | 0 (0 to 0) | -81·8 (-82·2 to -80·5) | 520·8 (155·5 to 1170·5) | 0·2 (0·1 to 0·5) | -100 (-100 to -100) |
|  | Southern Latin America | 19·5 (4·5 to 59·2) | 0 (0 to 0·1) | -80·7 (-81·7 to -81·1) | 1915·8 (367 to 6573·2) | 3·1 (0·6 to 10·4) | -100 (-100 to -100) |
|  | Caribbean | 1 (0·3 to 2·6) | 0 (0 to 0) | -85·1 (-85·7 to -85·8) | 107·2 (27 to 309·9) | 0·2 (0·1 to 0·7) | -100 (-100 to -100) |
|  | Central Europe | 0 (0 to 0) | 0 (0 to 0) | - | 0 (0 to 0) | 0 (0 to 0) | - |
|  | Eastern Europe | 0 (0 to 0) | 0 (0 to 0) | - | 0 (0 to 0) | 0 (0 to 0) | - |
|  | Central Asia | 0 (0 to 0) | 0 (0 to 0) | - | 0 (0 to 0) | 0 (0 to 0) | - |
|  | North Africa and Middle East | 181·4 (46·2 to 488) | 0 (0 to 0·1) | -91·6 (-92·2 to -92·7) | 23461·4 (4594·5 to 72318) | 3·6 (0·7 to 11·1) | -100 (-100 to -100) |
|  | South Asia | 0 (0 to 0) | 0 (0 to 0) | - | 0 (0 to 0) | 0 (0 to 0) | - |
|  | Southeast Asia | 0 (0 to 0) | 0 (0 to 0) | - | 0 (0 to 0) | 0 (0 to 0) | - |
|  | East Asia | 0 (0 to 0) | 0 (0 to 0) | - | 0 (0 to 0) | 0 (0 to 0) | - |
|  | Oceania | 0 (0 to 0) | 0 (0 to 0) | - | 0 (0 to 0) | 0 (0 to 0) | - |
|  | Western Sub-Saharan Africa | 1178·7 (461·7 to 2521·5) | 0·2 (0·1 to 0·5) | -91·2 (-91·1 to -91·4) | 160959·1 (60857·3 to 335271·2) | 27 (10·4 to 55·2) | -92·2 (-93·6 to -91·6) |
|  | Eastern Sub-Saharan Africa | 667·4 (234·5 to 1501·3) | 0·1 (0·1 to 0·3) | -86 (-87·2 to -86·3) | 93014·2 (31321·2 to 219841·9) | 18·7 (6·2 to 43·5) | -95·9 (-95·4 to -96) |
|  | Central Sub-Saharan Africa | 281·8 (94·4 to 681·1) | 0·2 (0·1 to 0·5) | -87·5 (-87·5 to -87·7) | 34204·2 (10029 to 88384·6) | 22·1 (6·5 to 57·6) | -100 (-100 to -100) |
|  | Southern Sub-Saharan Africa | 0 (0 to 0) | 0 (0 to 0) | - | 0 (0 to 0) | 0 (0 to 0) | - |
|  |  |  |  |  |  |  |  |
| **Rabies** | Global | 391·3 (233·6 to 549·3) | 0 (0 to 0) | -69·4 (-74·7 to -66·8) | 569550·3 (323361·7 to 828522·2) | 7·5 (4·2 to 11) | -92·6 (-94·4 to -91·4) |
|  | High-income Asia Pacific | 0 (0 to 0) | 0 (0 to 0) | -92·3 (-92·5 to -92) | 5·1 (4·2 to 6) | 0 (0 to 0) | 114·2 (94·9 to 145·4) |
|  | High-income North America | 0·3 (0·2 to 0·3) | 0 (0 to 0) | 181·5 (167 to 195·8) | 312·1 (278·7 to 350·4) | 0·1 (0·1 to 0·1) | 1079·8 (647·2 to 1751·1) |
|  | Western Europe | 0·1 (0·1 to 0·1) | 0 (0 to 0) | 100·4 (101·1 to 106·3) | 47·2 (40·3 to 53·2) | 0 (0 to 0) | 38392·2 (26909·9 to 55986) |
|  | Australasia | 0 (0 to 0) | 0 (0 to 0) | 37·7 (12·1 to 73·5) | 4 (2·6 to 6·3) | 0 (0 to 0) | 8449 (2006·6 to 18932) |
|  | Andean Latin America | 0 (0 to 0) | 0 (0 to 0) | -99·7 (-99·8 to -99·5) | 2·1 (0·9 to 3·8) | 0 (0 to 0) | -99·9 (-99·9 to -99·9) |
|  | Tropical Latin America | 0 (0 to 0) | 0 (0 to 0) | -99·6 (-99·6 to -99·7) | 19·9 (16·9 to 23·6) | 0 (0 to 0) | -99·9 (-99·9 to -99·9) |
|  | Central Latin America | 0 (0 to 0) | 0 (0 to 0) | -99·9 (-99·9 to -99·9) | 3·9 (3·3 to 5) | 0 (0 to 0) | -99·6 (-99·6 to -99·6) |
|  | Southern Latin America | 0 (0 to 0) | 0 (0 to 0) | -9·1 (-13·2 to -13·6) | 0·4 (0·3 to 0·5) | 0 (0 to 0) | 9829·7 (10200·7 to 9291·1) |
|  | Caribbean | 0 (0 to 0) | 0 (0 to 0) | -24·9 (-30·9 to -20) | 18·4 (6·9 to 37·6) | 0 (0 to 0·1) | 12377 (11772·9 to 12632·2) |
|  | Central Europe | 0 (0 to 0) | 0 (0 to 0) | -98·1 (-98·3 to -97·8) | 2·7 (2 to 3·6) | 0 (0 to 0) | 21471·1 (9517·7 to 40831·2) |
|  | Eastern Europe | 0·2 (0·2 to 0·3) | 0 (0 to 0) | -63·4 (-67·6 to -59·6) | 179 (143 to 225) | 0·1 (0·1 to 0·1) | -54 (-77 to -33·1) |
|  | Central Asia | 0·4 (0·2 to 0·5) | 0 (0 to 0) | -49·4 (-43·4 to -57·6) | 508·5 (303·7 to 844·1) | 0·5 (0·3 to 0·9) | -99·7 (-99·8 to -99·7) |
|  | North Africa and Middle East | 0·6 (0·3 to 0·9) | 0 (0 to 0) | -90·1 (-91·1 to -89·4) | 847·4 (393·3 to 1345·8) | 0·1 (0·1 to 0·2) | 798·3 (1011·3 to 748·6) |
|  | South Asia | 199·5 (132·8 to 272·3) | 0 (0 to 0) | -80·8 (-82·5 to -79) | 257449·3 (166294·8 to 365123·3) | 14·2 (9·1 to 20·2) | -100 (-100 to -100) |
|  | Southeast Asia | 25·5 (15·1 to 37·3) | 0 (0 to 0) | -70·1 (-74·6 to -67·4) | 36335·5 (21329·1 to 54988·2) | 5·4 (3·1 to 8·2) | -100 (-100 to -100) |
|  | East Asia | 24·1 (12·9 to 36·5) | 0 (0 to 0) | -63·3 (-65·8 to -60·3) | 26591·1 (14377·3 to 39906·1) | 1·8 (1 to 2·8) | 229·9 (124·2 to 271·3) |
|  | Oceania | 0·1 (0 to 0·2) | 0 (0 to 0) | -28·2 (-31·9 to -34·6) | 48·9 (11·5 to 138·9) | 0·6 (0·1 to 1·6) | 1051·9 (2062·9 to 440·7) |
|  | Western Sub-Saharan Africa | 65·1 (25·9 to 103·9) | 0 (0 to 0) | -45·3 (-46·6 to -52·3) | 119825·1 (47579·1 to 192226·9) | 19·8 (7·9 to 31·4) | -99·9 (-99·9 to -99·9) |
|  | Eastern Sub-Saharan Africa | 73·6 (28·4 to 162·3) | 0 (0 to 0) | -65·2 (-69·3 to -64·8) | 124148·3 (46424·9 to 272056·1) | 26·7 (10·3 to 59·7) | -100 (-100 to -100) |
|  | Central Sub-Saharan Africa | 0·8 (0·2 to 2·3) | 0 (0 to 0) | -41·7 (-31·3 to -48·7) | 1354 (272·3 to 4062·4) | 0·9 (0·2 to 2·5) | -99·8 (-99·3 to -99·9) |
|  | Southern Sub-Saharan Africa | 1·1 (0·7 to 1·8) | 0 (0 to 0) | -31·1 (-24·6 to -31·5) | 1847·5 (1101·2 to 3053) | 2·2 (1·3 to 3·7) | -82·5 (-91·7 to -67·9) |
|  |  |  |  |  |  |  |  |
| **Onchocerciasis** | Global | 19596822·3 (17757021·3 to 21711344·1) | 246·2 (222·7 to 273·1) | -44·6 (-45·6 to -43·8) | 1262988·1 (753578·3 to 1907363·1) | 15·8 (9·4 to 23·9) | -100 (-100 to -100) |
|  | High-income Asia Pacific | 0 (0 to 0) | 0 (0 to 0) | - | 0 (0 to 0) | 0 (0 to 0) | - |
|  | High-income North America | 0 (0 to 0) | 0 (0 to 0) | - | 0 (0 to 0) | 0 (0 to 0) | - |
|  | Western Europe | 0 (0 to 0) | 0 (0 to 0) | - | 0 (0 to 0) | 0 (0 to 0) | - |
|  | Australasia | 0 (0 to 0) | 0 (0 to 0) | - | 0 (0 to 0) | 0 (0 to 0) | - |
|  | Andean Latin America | 0 (0 to 0) | 0 (0 to 0) | -100 (-100 to -100) | 0 (0 to 0) | 0 (0 to 0) | -100 (-100 to -100) |
|  | Tropical Latin America | 219·7 (20·8 to 934·7) | 0·1 (0 to 0·4) | -99·3 (-99·9 to -97·3) | 17·9 (1·7 to 65·8) | 0 (0 to 0) | -100 (-100 to -100) |
|  | Central Latin America | 5040·1 (2324·8 to 8740·1) | 1·9 (0·9 to 3·4) | -99 (-99·5 to -98·3) | 451·3 (185·6 to 813·6) | 0·2 (0·1 to 0·3) | -94·8 (-95·6 to -94·6) |
|  | Southern Latin America | 0 (0 to 0) | 0 (0 to 0) | - | 0 (0 to 0) | 0 (0 to 0) | - |
|  | Caribbean | 0 (0 to 0) | 0 (0 to 0) | - | 0 (0 to 0) | 0 (0 to 0) | - |
|  | Central Europe | 0 (0 to 0) | 0 (0 to 0) | - | 0 (0 to 0) | 0 (0 to 0) | - |
|  | Eastern Europe | 0 (0 to 0) | 0 (0 to 0) | - | 0 (0 to 0) | 0 (0 to 0) | - |
|  | Central Asia | 0 (0 to 0) | 0 (0 to 0) | - | 0 (0 to 0) | 0 (0 to 0) | - |
|  | North Africa and Middle East | 70795·5 (64301·6 to 78520·5) | 11·6 (10·5 to 12·8) | -67·1 (-66·7 to -67·4) | 4371·3 (2588·8 to 6332·4) | 0·7 (0·4 to 1·1) | 4484·7 (4024·7 to 4739·1) |
|  | South Asia | 0 (0 to 0) | 0 (0 to 0) | - | 0 (0 to 0) | 0 (0 to 0) | - |
|  | Southeast Asia | 0 (0 to 0) | 0 (0 to 0) | - | 0 (0 to 0) | 0 (0 to 0) | - |
|  | East Asia | 0 (0 to 0) | 0 (0 to 0) | - | 0 (0 to 0) | 0 (0 to 0) | - |
|  | Oceania | 0 (0 to 0) | 0 (0 to 0) | - | 0 (0 to 0) | 0 (0 to 0) | - |
|  | Western Sub-Saharan Africa | 5277019·9 (4395464 to 6278936·9) | 1393·6 (1193·5 to 1636·7) | -81·6 (-82·8 to -80·5) | 379303·1 (206205·2 to 603104·4) | 106·5 (60·8 to 164) | -100 (-100 to -100) |
|  | Eastern Sub-Saharan Africa | 3043682·4 (2697822 to 3437058·4) | 890·3 (801·4 to 998·8) | -59·6 (-59·3 to -59·8) | 199341·9 (116278·2 to 299901·8) | 63 (38·2 to 95·3) | -100 (-100 to -100) |
|  | Central Sub-Saharan Africa | 11200064·8 (10468183·5 to 12057792·5) | 9857·7 (9222·9 to 10576·1) | -46·5 (-46·6 to -46·5) | 679502·5 (421054·2 to 1016962) | 627·2 (400·7 to 918·2) | -100 (-100 to -100) |
|  | Southern Sub-Saharan Africa | 0 (0 to 0) | 0 (0 to 0) | - | 0 (0 to 0) | 0 (0 to 0) | - |
|  |  |  |  |  |  |  |  |
| **Trachoma** | Global | 1414047·3 (1100377·4 to 1799564·3) | 16·4 (12·7 to 20·9) | -67·9 (-69 to -66·9) | 123190·2 (80954·1 to 174324·8) | 1·4 (0·9 to 2) | -98·3 (-98·6 to -98·1) |
|  | High-income Asia Pacific | 0 (0 to 0) | 0 (0 to 0) | - | 0 (0 to 0) | 0 (0 to 0) | - |
|  | High-income North America | 0 (0 to 0) | 0 (0 to 0) | - | 0 (0 to 0) | 0 (0 to 0) | - |
|  | Western Europe | 0 (0 to 0) | 0 (0 to 0) | - | 0 (0 to 0) | 0 (0 to 0) | - |
|  | Australasia | 75·5 (44·7 to 122·2) | 0·1 (0·1 to 0·2) | -29·2 (-35 to -21·3) | 6·6 (3·7 to 10·7) | 0 (0 to 0) | 5910·6 (7128 to 5127) |
|  | Andean Latin America | 154·1 (93·4 to 235·7) | 0·3 (0·2 to 0·4) | -86·6 (-86·9 to -86·9) | 18·9 (9·4 to 32·1) | 0 (0 to 0·1) | -96·2 (-95·8 to -96·6) |
|  | Tropical Latin America | 1854·9 (1251·9 to 2637·6) | 0·7 (0·5 to 1·1) | -66·5 (-66·4 to -68·5) | 274·7 (152·7 to 445·3) | 0·1 (0·1 to 0·2) | -100 (-100 to -100) |
|  | Central Latin America | 6497 (4925·1 to 8319·1) | 2·6 (2 to 3·3) | -60·9 (-58·7 to -62·4) | 611·4 (384·9 to 898) | 0·2 (0·2 to 0·4) | -100 (-100 to -100) |
|  | Southern Latin America | 0 (0 to 0) | 0 (0 to 0) | - | 0 (0 to 0) | 0 (0 to 0) | - |
|  | Caribbean | 0 (0 to 0) | 0 (0 to 0) | - | 0 (0 to 0) | 0 (0 to 0) | - |
|  | Central Europe | 0 (0 to 0) | 0 (0 to 0) | - | 0 (0 to 0) | 0 (0 to 0) | - |
|  | Eastern Europe | 0 (0 to 0) | 0 (0 to 0) | - | 0 (0 to 0) | 0 (0 to 0) | - |
|  | Central Asia | 0 (0 to 0) | 0 (0 to 0) | - | 0 (0 to 0) | 0 (0 to 0) | - |
|  | North Africa and Middle East | 144755·7 (104658 to 198215·9) | 34·7 (24·8 to 47·6) | -90·6 (-91·1 to -89·9) | 13996 (8445·1 to 21186) | 3·4 (2·1 to 5·1) | -90·1 (-90·7 to -89·5) |
|  | South Asia | 382944·3 (278772·7 to 515970) | 28·1 (20·5 to 37·4) | -58·7 (-59·3 to -57·4) | 35553·4 (22641·2 to 51615·7) | 2·7 (1·7 to 3·9) | -94·1 (-94·2 to -94·1) |
|  | Southeast Asia | 29711·9 (21980·1 to 38959·6) | 4·8 (3·6 to 6·1) | -87·9 (-88·1 to -87·9) | 2722·2 (1679·7 to 4078·5) | 0·5 (0·3 to 0·7) | -86·3 (-87·2 to -84·7) |
|  | East Asia | 12585·9 (8425·7 to 17662) | 0·6 (0·4 to 0·8) | -90·5 (-91·2 to -89·9) | 1725·9 (922·2 to 2836·3) | 0·1 (0 to 0·1) | -100 (-100 to -100) |
|  | Oceania | 1408·1 (948·6 to 2007·8) | 16·7 (11·5 to 22·9) | -82 (-82·2 to -82·8) | 141·2 (82·4 to 220·2) | 1·7 (1 to 2·7) | 384 (420·1 to 327·7) |
|  | Western Sub-Saharan Africa | 129944·5 (95631·4 to 168967·5) | 72·5 (53 to 95) | -79·2 (-80·8 to -78·1) | 14630·1 (8733 to 22345·7) | 8·3 (4·9 to 12·7) | -100 (-100 to -100) |
|  | Eastern Sub-Saharan Africa | 695940·3 (558638·5 to 863534·3) | 420·6 (339·7 to 518·7) | -51 (-53·4 to -48·7) | 52289 (35970·3 to 71823) | 31·4 (21·4 to 42·9) | -97·5 (-98 to -97·1) |
|  | Central Sub-Saharan Africa | 2198·6 (1549·4 to 2924·4) | 5·3 (3·7 to 7·1) | -85 (-85·3 to -85) | 240·9 (139·8 to 383·5) | 0·6 (0·3 to 0·9) | -92·3 (-91·9 to -92·5) |
|  | Southern Sub-Saharan Africa | 5976·5 (3763·5 to 9165·1) | 10·5 (6·8 to 15·7) | -42·2 (-43 to -40·3) | 979·9 (529·9 to 1608·5) | 1·7 (0·9 to 2·8) | -9·2 (5 to -19) |
|  |  |  |  |  |  |  |  |
| **Zika** | Global | 2935 (1172·3 to 7213) | 0 (0 to 0·1) | - | 157·9 (103·3 to 260·9) | 0 (0 to 0) | - |
|  | High-income Asia Pacific | 0 (0 to 0) | 0 (0 to 0) | - | 0 (0 to 0) | 0 (0 to 0) | - |
|  | High-income North America | 0 (0 to 0·1) | 0 (0 to 0) | - | 0 (0 to 0) | 0 (0 to 0) | - |
|  | Western Europe | 0 (0 to 0) | 0 (0 to 0) | - | 0 (0 to 0) | 0 (0 to 0) | - |
|  | Australasia | 0 (0 to 0) | 0 (0 to 0) | - | 0 (0 to 0) | 0 (0 to 0) | - |
|  | Andean Latin America | 517·2 (122·8 to 1724·7) | 0·8 (0·2 to 2·6) | - | 29·8 (16·5 to 55·6) | 0 (0 to 0·1) | - |
|  | Tropical Latin America | 1423·5 (611·5 to 3526) | 0·6 (0·3 to 1·5) | - | 75·3 (49·6 to 110·6) | 0 (0 to 0·1) | - |
|  | Central Latin America | 883·1 (200·4 to 3809·1) | 0·4 (0·1 to 1·5) | - | 48 (24·9 to 128·3) | 0 (0 to 0·1) | - |
|  | Southern Latin America | 1·8 (0·2 to 9·1) | 0 (0 to 0) | - | 0·1 (0 to 0·2) | 0 (0 to 0) | - |
|  | Caribbean | 109·4 (22·4 to 365·3) | 0·2 (0 to 0·8) | - | 4·6 (2·2 to 9·8) | 0 (0 to 0) | - |
|  | Central Europe | 0 (0 to 0) | 0 (0 to 0) | - | 0 (0 to 0) | 0 (0 to 0) | - |
|  | Eastern Europe | 0 (0 to 0) | 0 (0 to 0) | - | 0 (0 to 0) | 0 (0 to 0) | - |
|  | Central Asia | 0 (0 to 0) | 0 (0 to 0) | - | 0 (0 to 0) | 0 (0 to 0) | - |
|  | North Africa and Middle East | 0 (0 to 0) | 0 (0 to 0) | - | 0 (0 to 0) | 0 (0 to 0) | - |
|  | South Asia | 0 (0 to 0) | 0 (0 to 0) | - | 0 (0 to 0) | 0 (0 to 0) | - |
|  | Southeast Asia | 0 (0 to 0) | 0 (0 to 0) | - | 0 (0 to 0) | 0 (0 to 0) | - |
|  | East Asia | 0 (0 to 0) | 0 (0 to 0) | - | 0 (0 to 0) | 0 (0 to 0) | - |
|  | Oceania | 0 (0 to 0) | 0 (0 to 0) | - | 0 (0 to 0) | 0 (0 to 0) | - |
|  | Western Sub-Saharan Africa | 0 (0 to 0) | 0 (0 to 0) | - | 0 (0 to 0) | 0 (0 to 0) | - |
|  | Eastern Sub-Saharan Africa | 0 (0 to 0) | 0 (0 to 0) | - | 0 (0 to 0) | 0 (0 to 0) | - |
|  | Central Sub-Saharan Africa | 0 (0 to 0) | 0 (0 to 0) | - | 0 (0 to 0) | 0 (0 to 0) | - |
|  | Southern Sub-Saharan Africa | 0 (0 to 0) | 0 (0 to 0) | - | 0 (0 to 0) | 0 (0 to 0) | - |
|  |  |  |  |  |  |  |  |
| **Ebola** | Global | 62 (18·1 to 163·3) | 0 (0 to 0) | - | 2602·3 (2130·1 to 3062·4) | 0 (0 to 0) | - |
|  | High-income Asia Pacific | 0 (0 to 0) | 0 (0 to 0) | - | 0 (0 to 0) | 0 (0 to 0) | - |
|  | High-income North America | 0 (0 to 0) | 0 (0 to 0) | - | 0 (0 to 0) | 0 (0 to 0) | - |
|  | Western Europe | 0 (0 to 0) | 0 (0 to 0) | - | 0 (0 to 0) | 0 (0 to 0) | - |
|  | Australasia | 0 (0 to 0) | 0 (0 to 0) | - | 0 (0 to 0) | 0 (0 to 0) | - |
|  | Andean Latin America | 0 (0 to 0) | 0 (0 to 0) | - | 0 (0 to 0) | 0 (0 to 0) | - |
|  | Tropical Latin America | 0 (0 to 0) | 0 (0 to 0) | - | 0 (0 to 0) | 0 (0 to 0) | - |
|  | Central Latin America | 0 (0 to 0) | 0 (0 to 0) | - | 0 (0 to 0) | 0 (0 to 0) | - |
|  | Southern Latin America | 0 (0 to 0) | 0 (0 to 0) | - | 0 (0 to 0) | 0 (0 to 0) | - |
|  | Caribbean | 0 (0 to 0) | 0 (0 to 0) | - | 0 (0 to 0) | 0 (0 to 0) | - |
|  | Central Europe | 0 (0 to 0) | 0 (0 to 0) | - | 0 (0 to 0) | 0 (0 to 0) | - |
|  | Eastern Europe | 0 (0 to 0) | 0 (0 to 0) | - | 0 (0 to 0) | 0 (0 to 0) | - |
|  | Central Asia | 0 (0 to 0) | 0 (0 to 0) | - | 0 (0 to 0) | 0 (0 to 0) | - |
|  | North Africa and Middle East | 0 (0 to 0) | 0 (0 to 0) | - | 0 (0 to 0) | 0 (0 to 0) | - |
|  | South Asia | 0 (0 to 0) | 0 (0 to 0) | - | 0 (0 to 0) | 0 (0 to 0) | - |
|  | Southeast Asia | 0 (0 to 0) | 0 (0 to 0) | - | 0 (0 to 0) | 0 (0 to 0) | - |
|  | East Asia | 0 (0 to 0) | 0 (0 to 0) | - | 0 (0 to 0) | 0 (0 to 0) | - |
|  | Oceania | 0 (0 to 0) | 0 (0 to 0) | - | 0 (0 to 0) | 0 (0 to 0) | - |
|  | Western Sub-Saharan Africa | 19·8 (9·2 to 27·7) | 0 (0 to 0) | - | 1298·8 (1065·2 to 1529·3) | 0·3 (0·3 to 0·4) | - |
|  | Eastern Sub-Saharan Africa | 0 (0 to 0) | 0 (0 to 0) | - | 0 (0 to 0) | 0 (0 to 0) | - |
|  | Central Sub-Saharan Africa | 42·2 (8·9 to 137·6) | 0 (0 to 0·1) | - | 1303·5 (1064·9 to 1533·8) | 1·1 (0·9 to 1·3) | - |
|  | Southern Sub-Saharan Africa | 0 (0 to 0) | 0 (0 to 0) | - | 0 (0 to 0) | 0 (0 to 0) | - |
|  |  |  |  |  |  |  |  |
| **Intestinal nematode infections** | Global | 642720070·1 (586454625·3 to 714795829·9) | 8429·9 (7697·2 to 9362·2) | -30·3 (-40·2 to -20·3) | 1381641 (920413·4 to 2019784·4) | 18·8 (12·6 to 27·3) | -87·9 (-90 to -85·6) |
|  | High-income Asia Pacific | 2536045·6 (1561524 to 3856334·8) | 1834·1 (1122·7 to 2796·6) | -100 (-100 to -100) | 27·8 (15 to 51·7) | 0 (0 to 0) | 819 (929·7 to 894·9) |
|  | High-income North America | 0 (0 to 0) | 0 (0 to 0) | - | 0 (0 to 0) | 0 (0 to 0) | - |
|  | Western Europe | 0 (0 to 0) | 0 (0 to 0) | - | 0 (0 to 0) | 0 (0 to 0) | - |
|  | Australasia | 0 (0 to 0) | 0 (0 to 0) | - | 0 (0 to 0) | 0 (0 to 0) | - |
|  | Andean Latin America | 20139043·3 (17451511·4 to 22818555·5) | 30153·7 (26076·8 to 34143·5) | -28·2 (-29 to -26·3) | 25678·8 (15407·3 to 40933·8) | 38·6 (23·3 to 61·3) | -69·2 (-73·7 to -65·7) |
|  | Tropical Latin America | 26782784·8 (19230575·3 to 36503227·5) | 12453·3 (8957·6 to 16960·8) | -19·4 (-22·3 to -18·4) | 26280·9 (14600·8 to 46109·9) | 12·6 (7 to 22·1) | -100 (-100 to -100) |
|  | Central Latin America | 38819808·7 (33156911 to 46254923·9) | 15438 (13198·4 to 18381·6) | -62·4 (-64·2 to -60·1) | 45705·8 (26387·7 to 75174·9) | 18·4 (10·7 to 30·1) | -96·8 (-97·4 to -96·5) |
|  | Southern Latin America | 3934153·4 (2932509·2 to 5211520·9) | 6152·1 (4583·1 to 8150·8) | -100 (-100 to -100) | 578·6 (312·6 to 1019·9) | 0·9 (0·5 to 1·6) | 96 (131·2 to 74·7) |
|  | Caribbean | 4818849·2 (4120330·5 to 5667983·6) | 10407·6 (8906 to 12226·4) | 3·4 (0·1 to 6·2) | 8001·4 (5004·2 to 12609·2) | 17·6 (11·1 to 27·4) | -100 (-100 to -100) |
|  | Central Europe | 0 (0 to 0) | 0 (0 to 0) | - | 0 (0 to 0) | 0 (0 to 0) | - |
|  | Eastern Europe | 0 (0 to 0) | 0 (0 to 0) | - | 0 (0 to 0) | 0 (0 to 0) | - |
|  | Central Asia | 3414465·7 (2798826·6 to 4300175) | 3574·3 (2931·3 to 4498) | -9·9 (-11·3 to -9·2) | 2041·1 (1256·5 to 3194·8) | 2·1 (1·3 to 3·3) | 1061·3 (1208·6 to 924·6) |
|  | North Africa and Middle East | 42919222·8 (38002553·3 to 47848964·9) | 6696·7 (5925·2 to 7471·3) | 95·9 (104·8 to 91·5) | 18931·8 (12122 to 30733·5) | 3 (1·9 to 4·8) | -69·9 (-72·3 to -65·3) |
|  | South Asia | 189311941·2 (138471409 to 257370412·9) | 10033·4 (7313·9 to 13663·1) | -85·3 (-85·1 to -85·4) | 304601·7 (173711 to 508694·1) | 16·5 (9·5 to 27·4) | -74·3 (-76·1 to -72·8) |
|  | Southeast Asia | 51018141·7 (42810799·4 to 61248001) | 7444·3 (6249·9 to 8931·4) | -92·1 (-92·9 to -90·7) | 99590·6 (61650·9 to 154966·4) | 14·7 (9·3 to 22·7) | -100 (-100 to -100) |
|  | East Asia | 64162871 (43223153·9 to 92045034·8) | 5024·4 (3374·1 to 7233·9) | -75 (-71 to -77·6) | 23317·3 (11964·8 to 38808·1) | 1·8 (0·9 to 2·9) | -79·7 (-74·8 to -81·7) |
|  | Oceania | 2860296·8 (2269508·7 to 3521144·9) | 19320·1 (15333 to 23817·1) | -88·4 (-91·1 to -85) | 14454·4 (8911·7 to 21912·5) | 93·4 (57·1 to 143·7) | -76 (-74·6 to -76·4) |
|  | Western Sub-Saharan Africa | 74059698·7 (69338811·2 to 78417228·5) | 13401·8 (12561·7 to 14194·3) | -74·3 (-80·7 to -66) | 358743·5 (248202·2 to 494766·2) | 56·4 (38·9 to 79·2) | -64·3 (-70·4 to -58·8) |
|  | Eastern Sub-Saharan Africa | 73903152·4 (68268583·6 to 80449989·4) | 15446·4 (14245·5 to 16831·8) | -70·5 (-70·8 to -70·2) | 318718 (208914·2 to 472132·1) | 63·3 (41·2 to 95) | -94·9 (-95·5 to -94·4) |
|  | Central Sub-Saharan Africa | 34372253·5 (29552847·6 to 39143022·5) | 22494·6 (19311·1 to 25719·5) | -53·3 (-57·1 to -50·3) | 108034·7 (71684·1 to 161348·2) | 65·5 (43·1 to 98·7) | -73·1 (-74·3 to -70·1) |
|  | Southern Sub-Saharan Africa | 9667341·3 (7978872·9 to 11697668) | 11665·8 (9619·5 to 14101·6) | -74·5 (-76·8 to -71·5) | 26934·5 (15970·1 to 43969·8) | 32·7 (19·4 to 53·3) | -99 (-99 to -99) |
|  |  |  |  |  |  |  |  |
| **Leprosy** | Global | 408775·8 (357208·8 to 472239·3) | 4·8 (4·2 to 5·6) | -58·7 (-58·9 to -58·4) | 21427·5 (14005·5 to 31592·8) | 0·3 (0·2 to 0·4) | -73·2 (-74·3 to -72·6) |
|  | High-income Asia Pacific | 371·7 (283·7 to 477·2) | 0·1 (0·1 to 0·2) | -53·7 (-56 to -51·9) | 24 (14·2 to 36·8) | 0 (0 to 0) | 3401·7 (3563 to 3328·5) |
|  | High-income North America | 0 (0 to 0) | 0 (0 to 0) | - | 0 (0 to 0) | 0 (0 to 0) | - |
|  | Western Europe | 0 (0 to 0) | 0 (0 to 0) | - | 0 (0 to 0) | 0 (0 to 0) | - |
|  | Australasia | 3 (2·2 to 4·1) | 0 (0 to 0) | -26·9 (-27·7 to -25·5) | 0·1 (0 to 0·2) | 0 (0 to 0) | -23·9 (-31·4 to -16·8) |
|  | Andean Latin America | 476·9 (392·1 to 568·9) | 0·7 (0·6 to 0·9) | -80·6 (-81·2 to -80·3) | 24·7 (14·7 to 38·1) | 0 (0 to 0·1) | -69·4 (-70·6 to -69·2) |
|  | Tropical Latin America | 70291·1 (60713·6 to 80822) | 27·4 (23·7 to 31·4) | -30·2 (-30·8 to -30) | 3467·5 (2256·6 to 5069·2) | 1·3 (0·9 to 2) | -97·9 (-98 to -97·8) |
|  | Central Latin America | 3485·3 (2893·2 to 4098·8) | 1·3 (1·1 to 1·6) | -63·6 (-63·9 to -63·6) | 197·3 (121·5 to 298) | 0·1 (0 to 0·1) | 201·6 (197·9 to 194·9) |
|  | Southern Latin America | 581 (485·6 to 690·7) | 0·7 (0·6 to 0·9) | -50·7 (-51·1 to -49·3) | 30·4 (18·6 to 46·7) | 0 (0 to 0·1) | 869·5 (960·4 to 829·8) |
|  | Caribbean | 1482·6 (1263·3 to 1720·9) | 2·8 (2·4 to 3·3) | -60·3 (-60·7 to -60·4) | 81·1 (51 to 122) | 0·2 (0·1 to 0·2) | -97·9 (-98 to -97·8) |
|  | Central Europe | 0 (0 to 0) | 0 (0 to 0) | - | 0 (0 to 0) | 0 (0 to 0) | - |
|  | Eastern Europe | 0 (0 to 0) | 0 (0 to 0) | - | 0 (0 to 0) | 0 (0 to 0) | - |
|  | Central Asia | 482·3 (398·2 to 586·9) | 0·5 (0·4 to 0·6) | -39·4 (-39·5 to -38·3) | 13·2 (7·9 to 20·7) | 0 (0 to 0) | -100 (-100 to -100) |
|  | North Africa and Middle East | 4258·5 (3606·1 to 5018·8) | 0·7 (0·6 to 0·9) | -68·9 (-69·2 to -68·6) | 231·6 (142·4 to 349·6) | 0 (0 to 0·1) | -82·2 (-83·8 to -81·4) |
|  | South Asia | 208274·5 (180566·2 to 241187·3) | 12·2 (10·6 to 14·1) | -72·1 (-72·4 to -72) | 10954·9 (7117·4 to 16166·9) | 0·6 (0·4 to 0·9) | -100 (-100 to -100) |
|  | Southeast Asia | 52422 (45701·3 to 60330·9) | 7·2 (6·3 to 8·3) | -54·1 (-53·6 to -54·2) | 2711·8 (1766·6 to 3995·2) | 0·4 (0·2 to 0·5) | -51·6 (-51·8 to -51·4) |
|  | East Asia | 7593·5 (5942·6 to 9483·8) | 0·4 (0·3 to 0·5) | -73·3 (-74·6 to -72) | 445·1 (257·8 to 684·6) | 0 (0 to 0) | -9·5 (-9·3 to -10·5) |
|  | Oceania | 1432·5 (1263·8 to 1643·6) | 13·9 (12·3 to 15·7) | -51·8 (-50·7 to -52·7) | 73 (47·4 to 107·2) | 0·7 (0·5 to 1) | -83·5 (-83·5 to -83·1) |
|  | Western Sub-Saharan Africa | 21231·8 (18523·4 to 24641·1) | 7·3 (6·4 to 8·5) | -64·6 (-64·4 to -64·6) | 1166·3 (737·5 to 1733·7) | 0·4 (0·3 to 0·6) | -98·7 (-98·8 to -98·6) |
|  | Eastern Sub-Saharan Africa | 26413·4 (23563·1 to 29816·5) | 10·5 (9·4 to 11·8) | -69 (-68·1 to -70·2) | 1452·7 (936·5 to 2138·5) | 0·6 (0·4 to 0·8) | -100 (-100 to -100) |
|  | Central Sub-Saharan Africa | 9271·4 (8110·7 to 10668·2) | 11·3 (10 to 12·9) | -82·1 (-81·7 to -82·3) | 508·6 (318·6 to 757·4) | 0·6 (0·4 to 0·9) | -89 (-89·1 to -88·9) |
|  | Southern Sub-Saharan Africa | 704·4 (558·7 to 873·1) | 1 (0·8 to 1·2) | -43·5 (-44·5 to -43·1) | 45·1 (27·2 to 68·3) | 0·1 (0 to 0·1) | 407·1 (431·4 to 394·4) |
|  |  |  |  |  |  |  |  |
| **Guinea worm disease** | Global | 23·9 (18 to 31) | 0 (0 to 0) | -100 (-100 to -100) | 0·9 (0·5 to 1·4) | 0 (0 to 0) | -100 (-100 to -100) |
|  | High-income Asia Pacific | 0 (0 to 0) | 0 (0 to 0) | - | 0 (0 to 0) | 0 (0 to 0) | - |
|  | High-income North America | 0 (0 to 0) | 0 (0 to 0) | - | 0 (0 to 0) | 0 (0 to 0) | - |
|  | Western Europe | 0 (0 to 0) | 0 (0 to 0) | - | 0 (0 to 0) | 0 (0 to 0) | - |
|  | Australasia | 0 (0 to 0) | 0 (0 to 0) | - | 0 (0 to 0) | 0 (0 to 0) | - |
|  | Andean Latin America | 0 (0 to 0) | 0 (0 to 0) | - | 0 (0 to 0) | 0 (0 to 0) | - |
|  | Tropical Latin America | 0 (0 to 0) | 0 (0 to 0) | - | 0 (0 to 0) | 0 (0 to 0) | - |
|  | Central Latin America | 0 (0 to 0) | 0 (0 to 0) | - | 0 (0 to 0) | 0 (0 to 0) | - |
|  | Southern Latin America | 0 (0 to 0) | 0 (0 to 0) | - | 0 (0 to 0) | 0 (0 to 0) | - |
|  | Caribbean | 0 (0 to 0) | 0 (0 to 0) | - | 0 (0 to 0) | 0 (0 to 0) | - |
|  | Central Europe | 0 (0 to 0) | 0 (0 to 0) | - | 0 (0 to 0) | 0 (0 to 0) | - |
|  | Eastern Europe | 0 (0 to 0) | 0 (0 to 0) | - | 0 (0 to 0) | 0 (0 to 0) | - |
|  | Central Asia | 0 (0 to 0) | 0 (0 to 0) | - | 0 (0 to 0) | 0 (0 to 0) | - |
|  | North Africa and Middle East | 0 (0 to 0) | 0 (0 to 0) | -100 (-100 to -100) | 0 (0 to 0) | 0 (0 to 0) | -100 (-100 to -100) |
|  | South Asia | 0 (0 to 0) | 0 (0 to 0) | -100 (-100 to -100) | 0 (0 to 0) | 0 (0 to 0) | -100 (-100 to -100) |
|  | Southeast Asia | 0 (0 to 0) | 0 (0 to 0) | - | 0 (0 to 0) | 0 (0 to 0) | - |
|  | East Asia | 0 (0 to 0) | 0 (0 to 0) | - | 0 (0 to 0) | 0 (0 to 0) | - |
|  | Oceania | 0 (0 to 0) | 0 (0 to 0) | - | 0 (0 to 0) | 0 (0 to 0) | - |
|  | Western Sub-Saharan Africa | 22 (16·5 to 28·5) | 0 (0 to 0) | -100 (-100 to -100) | 0·8 (0·4 to 1·3) | 0 (0 to 0) | -100 (-100 to -100) |
|  | Eastern Sub-Saharan Africa | 1·9 (0·5 to 4·3) | 0 (0 to 0) | -100 (-100 to -100) | 0·1 (0 to 0·2) | 0 (0 to 0) | -100 (-100 to -100) |
|  | Central Sub-Saharan Africa | 0 (0 to 0) | 0 (0 to 0) | -100 (-100 to -100) | 0 (0 to 0) | 0 (0 to 0) | -100 (-100 to -100) |
|  | Southern Sub-Saharan Africa | 0 (0 to 0) | 0 (0 to 0) | - | 0 (0 to 0) | 0 (0 to 0) | - |
|  |  |  |  |  |  |  |  |
| **Other neglected tropical diseases** | Global | 103762442·3 (102134927·5 to 105436286·8) | 1381·2 (1358·6 to 1403·5) | -8·2 (-8·4 to -8) | 4218302·2 (3095236·3 to 5602012·8) | 58·4 (42·9 to 77·3) | -89·2 (-88·9 to -89·2) |
|  | High-income Asia Pacific | 582886·9 (502835·6 to 688862·9) | 341·2 (285·2 to 408·8) | -34·1 (-34·6 to -34·3) | 8895·4 (6087·2 to 12846·6) | 4·5 (2·9 to 7) | 219·6 (255·8 to 185·6) |
|  | High-income North America | 942285·2 (857141·4 to 1065707·7) | 269·5 (242·5 to 304·5) | -5 (-6·9 to -2·2) | 24291·9 (19118·3 to 31629·3) | 6·6 (5·1 to 8·8) | 605·5 (610·1 to 571·6) |
|  | Western Europe | 1040154·1 (963895·2 to 1156707·6) | 288·3 (264·8 to 321·4) | -30·3 (-29·2 to -31) | 20160·1 (14447·8 to 28264·3) | 5·6 (3·9 to 8·1) | -49·6 (-53·5 to -44·7) |
|  | Australasia | 80384·2 (67424·9 to 110552·3) | 301·4 (238·5 to 460·8) | -27·8 (-25·4 to -29·2) | 1197·6 (771·5 to 1875·1) | 4·2 (2·6 to 6·8) | 39·4 (42·8 to 42·5) |
|  | Andean Latin America | 595268·4 (537620·7 to 691497·6) | 912·5 (825·5 to 1064·7) | -39·1 (-38·9 to -37·1) | 16531·4 (11710·8 to 23156·2) | 25·8 (18·3 to 36) | -79·1 (-79·9 to -78·5) |
|  | Tropical Latin America | 2574599·2 (2329408·6 to 2886456·2) | 1201·4 (1087·7 to 1361·7) | -23·2 (-23·6 to -21·1) | 94761·2 (70177·3 to 127626·4) | 45·7 (33·3 to 61·6) | -22 (-35·3 to -15·4) |
|  | Central Latin America | 1427310·8 (1379784·7 to 1493949) | 602·1 (582·3 to 629·4) | -24·7 (-24·1 to -24·7) | 51730·7 (39672 to 67775·7) | 22·4 (17·1 to 29·4) | 217·1 (149·6 to 253·2) |
|  | Southern Latin America | 319835·5 (213310·4 to 483046·6) | 559·5 (366·7 to 860·4) | -28·3 (-37·5 to -20) | 4826·9 (3233·1 to 7566·7) | 8·1 (5·3 to 12·8) | 126·3 (158·9 to 92·6) |
|  | Caribbean | 756547·3 (711289·6 to 809762·3) | 1702·5 (1595·7 to 1825·5) | 3 (1·4 to 4·9) | 20873·7 (14015·9 to 29347·1) | 47·6 (31·8 to 67·1) | -89·2 (-89 to -88·8) |
|  | Central Europe | 832239·4 (789060·2 to 886499·6) | 872 (819·7 to 940·1) | -31·9 (-32·4 to -30·6) | 15934·9 (10816·5 to 22922·8) | 16·5 (11 to 24) | 15·2 (21·6 to 12·5) |
|  | Eastern Europe | 1462507·1 (1354146 to 1587484·8) | 786·2 (723·9 to 858·8) | -22·8 (-24·5 to -21) | 37473·4 (26968·5 to 53204·1) | 20·9 (14·9 to 29·3) | -86·2 (-88 to -84·1) |
|  | Central Asia | 1688745·1 (1592946·6 to 1817739·7) | 1727·1 (1628·3 to 1861) | -14·8 (-15·1 to -13·4) | 52289 (37094·6 to 74046·7) | 53·4 (37·9 to 75·8) | 20 (23·7 to 17·7) |
|  | North Africa and Middle East | 7441711·1 (7182492·5 to 7746681·5) | 1177·3 (1136·9 to 1224·4) | -25·3 (-25·1 to -25·6) | 221439·8 (156653·7 to 311003·4) | 35·2 (25 to 49·3) | 43 (48·4 to 35·7) |
|  | South Asia | 45892624 (44755328·9 to 47170863·6) | 2532·1 (2470 to 2599·4) | -11·3 (-11·6 to -10·8) | 1442285·6 (1018059·3 to 2034658·6) | 80·8 (57·3 to 114·1) | -38·6 (-41·9 to -38·2) |
|  | Southeast Asia | 7667517·8 (7327185·5 to 7987321·4) | 1137·7 (1088·8 to 1184·8) | -26·6 (-26·7 to -26·7) | 235104·4 (171115 to 319601·6) | 35·8 (26 to 48·5) | -1·9 (-8·5 to 0·7) |
|  | East Asia | 5649957·6 (5435305·2 to 5848651) | 403·3 (387·6 to 417·5) | -54·9 (-55·3 to -54·7) | 155565·4 (112376·9 to 213838) | 11·3 (8·1 to 15·6) | 58·1 (57·4 to 58·3) |
|  | Oceania | 265559·3 (228888·3 to 311763·8) | 1741·7 (1517·6 to 2024) | -6·8 (-10·9 to -3·4) | 6120·8 (3961·4 to 9697·2) | 42·9 (28·8 to 65·2) | -8 (-7·1 to -0·6) |
|  | Western Sub-Saharan Africa | 12741642·4 (12106877·6 to 13462583) | 2193·4 (2101·9 to 2297·8) | 7·4 (8·4 to 6·6) | 1355733·4 (922780·2 to 1768850·8) | 203·1 (141·7 to 260·9) | -76 (-78·1 to -75·2) |
|  | Eastern Sub-Saharan Africa | 8318792·4 (7890955·9 to 8731611·7) | 1686·9 (1614·1 to 1759·5) | -13·5 (-14·1 to -12·8) | 316515·2 (219403·1 to 440734·6) | 65·6 (44 to 91·2) | -74·4 (-71·5 to -74·9) |
|  | Central Sub-Saharan Africa | 2227573·8 (1999207·8 to 2499991·1) | 1461·2 (1328·6 to 1627·1) | -27·1 (-27·9 to -25·5) | 80172·8 (53436·5 to 113518·6) | 54·4 (34·8 to 76·2) | -79 (-78·8 to -77·3) |
|  | Southern Sub-Saharan Africa | 1254300·9 (1180643·5 to 1343008·8) | 1520·1 (1434 to 1623·4) | -10 (-10·2 to -9·8) | 56398·8 (40592·3 to 73957·3) | 69·5 (50·3 to 90·8) | -20·7 (-22·4 to -21·6) |
|  |  |  |  |  |  |  |  |
| **Food-borne trematodiases** | Global | 44466329·4 (40017217·6 to 50034921·1) | 526·7 (473·7 to 593·3) | -50·1 (-50·1 to -50·1) | 998028·5 (569766·4 to 1638112·2) | 11·8 (6·7 to 19·5) | -100 (-100 to -100) |
|  | High-income Asia Pacific | 1974178 (1750147·4 to 2202859·9) | 821·5 (731·3 to 915·2) | 11 (3 to 18·6) | 34442·6 (22551 to 50013·1) | 14·3 (9·2 to 20·2) | -99·3 (-99·5 to -99·2) |
|  | High-income North America | 0 (0 to 0) | 0 (0 to 0) | - | 0 (0 to 0) | 0 (0 to 0) | - |
|  | Western Europe | 100663·4 (77153·6 to 128320·8) | 18·2 (13·8 to 23·2) | -47·4 (-51·5 to -44·1) | 2043·7 (532·5 to 4030·8) | 0·4 (0·1 to 0·7) | 1914·9 (5126·4 to 1346) |
|  | Australasia | 0 (0 to 0) | 0 (0 to 0) | - | 0 (0 to 0) | 0 (0 to 0) | - |
|  | Andean Latin America | 1728083·3 (1412538 to 2105541·5) | 2606·1 (2127·3 to 3170·8) | -27·1 (-32·9 to -21·2) | 37253·5 (12655·2 to 70510·6) | 56·1 (19 to 106·1) | -46·8 (-34 to -46·6) |
|  | Tropical Latin America | 0 (0 to 0) | 0 (0 to 0) | - | 0 (0 to 0) | 0 (0 to 0) | - |
|  | Central Latin America | 0 (0 to 0) | 0 (0 to 0) | - | 0 (0 to 0) | 0 (0 to 0) | - |
|  | Southern Latin America | 0 (0 to 0) | 0 (0 to 0) | - | 0 (0 to 0) | 0 (0 to 0) | - |
|  | Caribbean | 0 (0 to 0) | 0 (0 to 0) | - | 0 (0 to 0) | 0 (0 to 0) | - |
|  | Central Europe | 0 (0 to 0) | 0 (0 to 0) | - | 0 (0 to 0) | 0 (0 to 0) | - |
|  | Eastern Europe | 1803863 (1560285·5 to 2077161·9) | 685·3 (601·8 to 780·6) | 0·4 (0·7 to 0·1) | 43222·6 (21534·3 to 70261) | 15·6 (7·9 to 24·7) | -100 (-100 to -100) |
|  | Central Asia | 3549·9 (3073·5 to 4087·4) | 3·8 (3·3 to 4·3) | -91·1 (-90·9 to -90·7) | 84·3 (42·1 to 135·2) | 0·1 (0 to 0·1) | -63·2 (-80·4 to -53·7) |
|  | North Africa and Middle East | 830731·4 (639516·5 to 1050045·7) | 135·8 (106·5 to 171) | -35·7 (-40 to -30·1) | 16951·2 (4460·7 to 33949·2) | 2·8 (0·7 to 5·5) | 204·6 (641 to 130·1) |
|  | South Asia | 0 (0 to 0) | 0 (0 to 0) | - | 0 (0 to 0) | 0 (0 to 0) | - |
|  | Southeast Asia | 4708037·8 (4280245·9 to 5159477·8) | 652 (593·4 to 711·7) | -75 (-74·6 to -75·3) | 95733·3 (58765·9 to 138444) | 13·1 (8·2 to 19·1) | -100 (-100 to -100) |
|  | East Asia | 33317222·6 (29251038·7 to 38353602) | 1862·4 (1640·7 to 2161·6) | -41·6 (-40·2 to -42·2) | 768297·4 (383882·8 to 1367826·1) | 42·6 (21·3 to 77) | -100 (-100 to -100) |
|  | Oceania | 0 (0 to 0) | 0 (0 to 0) | - | 0 (0 to 0) | 0 (0 to 0) | - |
|  | Western Sub-Saharan Africa | 0 (0 to 0) | 0 (0 to 0) | - | 0 (0 to 0) | 0 (0 to 0) | - |
|  | Eastern Sub-Saharan Africa | 0 (0 to 0) | 0 (0 to 0) | - | 0 (0 to 0) | 0 (0 to 0) | - |
|  | Central Sub-Saharan Africa | 0 (0 to 0) | 0 (0 to 0) | - | 0 (0 to 0) | 0 (0 to 0) | - |
|  | Southern Sub-Saharan Africa | 0 (0 to 0) | 0 (0 to 0) | - | 0 (0 to 0) | 0 (0 to 0) | - |

**Table S1** Prevalence and disability-adjusted life-years (DALYs) for NTDs, malaria, and diseases comprising NTDs in 2021, and percentage change in age-standardized rates (ASRs) per 100,000 population from 1990 to 2021, by Global Burden of Disease region. These data were generated using data provided by <https://ghdx.healthdata.org/gbd-results-tool.>

| **year** | **NTDs** | | | | | | **Malaria** | | | | | |
| --- | --- | --- | --- | --- | --- | --- | --- | --- | --- | --- | --- | --- |
|  | **Female** | | | **Male** | | | **Male** | | | **Female** | | |
|  | Rate | lower | upper | Rate | lower | upper | Rate | lower | upper | Rate | lower | upper |
| 2022 | 201.5776 | 194.2383 | 208.917 | 215.91833 | 205.91896 | 225.9177 | 835.4407 | 801.6859 | 869.1955 | 798.467 | 766.2557 | 830.6782 |
| 2023 | 196.8017 | 186.8724 | 206.731 | 207.74668 | 190.87913 | 224.6142 | 844.2283 | 802.1888 | 886.2678 | 791.6595 | 755.1148 | 828.2043 |
| 2024 | 191.8795 | 179.9622 | 203.7968 | 198.53764 | 176.02585 | 221.0494 | 835.0793 | 784.3201 | 885.8384 | 766.237 | 725.4384 | 807.0356 |
| 2025 | 186.9114 | 173.4181 | 200.4046 | 188.67179 | 161.58214 | 215.7614 | 806.0081 | 744.4312 | 867.5849 | 718.646 | 670.6356 | 766.6564 |
| 2026 | 181.9402 | 167.1606 | 196.7198 | 178.39061 | 147.62381 | 209.1574 | 759.1046 | 686.5286 | 831.6806 | 656.3658 | 601.3289 | 711.4027 |
| 2027 | 176.9856 | 161.1366 | 192.8346 | 167.84341 | 134.1376 | 201.5492 | 698.7095 | 616.7533 | 780.6658 | 587.7845 | 526.2239 | 649.3451 |
| 2028 | 172.0553 | 155.3046 | 188.806 | 157.12103 | 121.0692 | 193.1729 | 631.1865 | 542.1919 | 720.181 | 520.5823 | 452.6651 | 588.4995 |
| 2029 | 167.1515 | 149.6318 | 184.6712 | 146.27758 | 108.35046 | 184.2047 | 564.0291 | 469.7749 | 658.2833 | 462.6321 | 387.6387 | 537.6255 |
| 2030 | 162.2738 | 144.0919 | 180.4556 | 135.34424 | 95.91305 | 174.7754 | 504.8255 | 405.5193 | 604.1317 | 420.9016 | 336.9117 | 504.8914 |
| 2031 | 157.4204 | 138.6637 | 176.1771 | 124.33802 | 83.69458 | 164.9815 | 460.1698 | 354.266 | 566.0737 | 399.7238 | 304.7025 | 494.7452 |
| 2032 | 152.5894 | 133.3302 | 171.8486 | 113.2672 | 71.64072 | 154.8937 | 434.7065 | 319.8418 | 549.5713 | 399.9286 | 293.1606 | 506.6966 |
| 2033 | 147.7784 | 128.077 | 167.4797 | 102.13483 | 59.70546 | 144.5642 | 430.445 | 304.9256 | 555.9644 | 418.7918 | 301.4458 | 536.1379 |
| 2034 | 142.9851 | 122.8926 | 163.0775 | 90.94084 | 47.8504 | 134.0313 | 446.4443 | 310.2554 | 582.6332 | 450.4974 | 325.2098 | 575.785 |
| 2035 | 138.2074 | 117.7671 | 158.6477 | 79.68338 | 36.0438 | 123.323 | 478.9158 | 333.7666 | 624.065 | 487.0574 | 356.8807 | 617.2342 |
| 2036 | 133.4431 | 112.692 | 154.1943 | 68.35965 | 24.25952 | 112.4598 | 521.7324 | 370.3194 | 673.1453 | 519.6216 | 386.7545 | 652.4887 |

|  |
| --- |

**Table S2** VAR Model's Projections for the Age-standardized Disability-Adjusted Life Years (DALYs) Rates of Malaria and Neglected Tropical Diseases (NTDs) from 2022 to 2036, along with the 95% Confidence Intervals.

| Lag Order (p) | AIC | BIC | HQ | RMSE | Residual Autocorrelation |
| --- | --- | --- | --- | --- | --- |
| 1 | -488.43 | -459.75 | -26.81 | 0.3008 | Present |
| **2** | **-526.37** | **-475.93** | **-28.36** | 0.3520 | **Absent** |
| 3 | -534.59 | -463.49 | -29.02 | 0.3727 | Absent |
| 4 | -527.45 | -436.86 | -29.20 | 0.3984 | Present |

**Table S3** Comparison of VAR Models with Different Lag Orders

| **Evaluation Metrics** | **Malaria** | | **NTDs** | |
| --- | --- | --- | --- | --- |
|  | **Male** | **Female** | **Male** | **Female** |
| MSE | 0.1239153 | 0.08789275 | 0.005858089 | 0.01457981 |
| RMSE | 0.352016 | 0.2964671 | 0.07653815 | 0.1207469 |

**Table S4** In the rolling model validation for assessing the predictive performance of the RVA model, given the dataset spanning from 1990 to 2021 with a total of 32 data points, and with a lag of 2 selected for all, the degrees of freedom would be 30. Using 80% of the degrees of freedom as the training set, and the next data point as the test set, a VAR model is trained for each training set, and the subsequent data point is predicted. The squared errors between the predicted values and the actual values are calculated and stored.


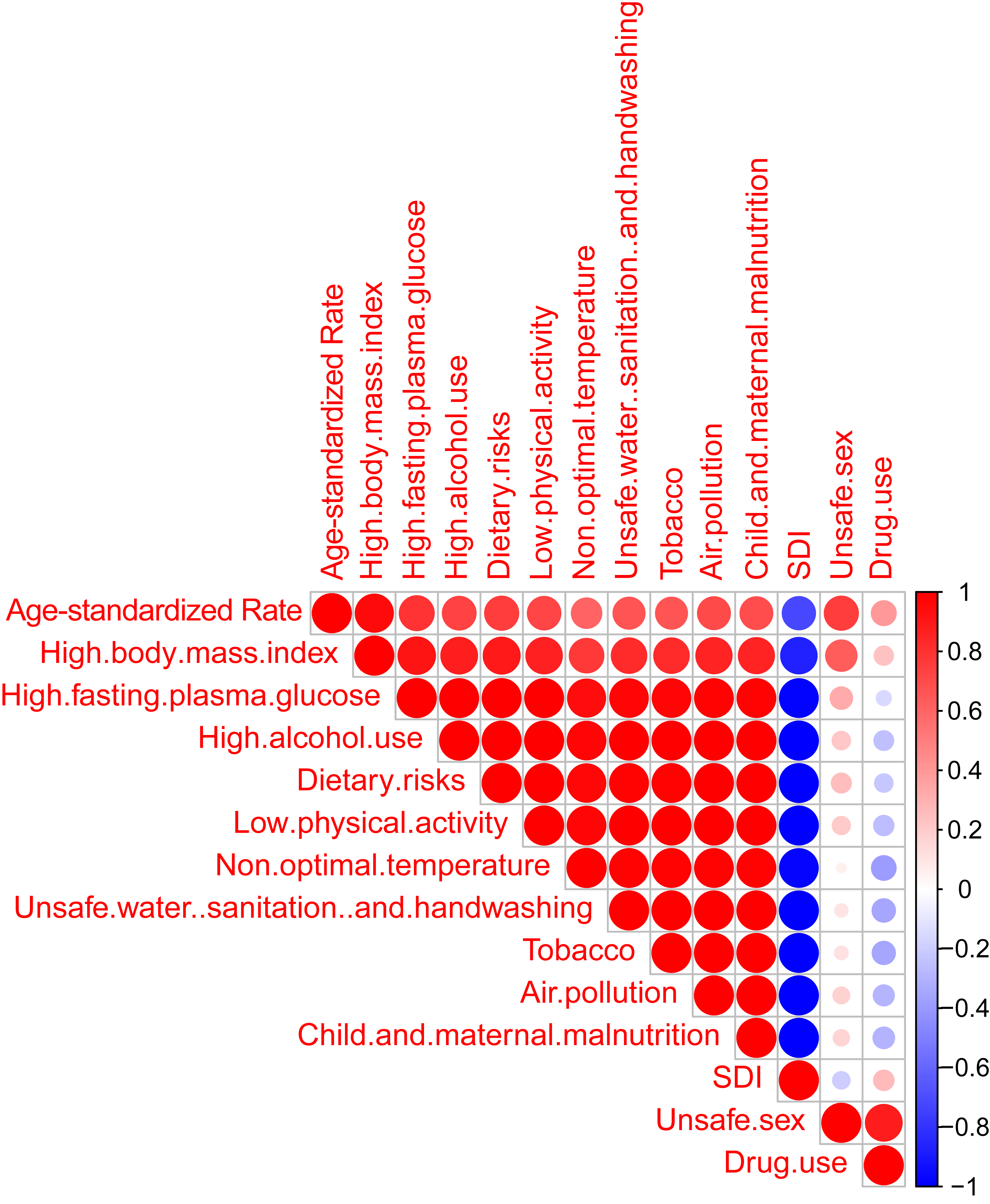


Fig.S1 The correlation heatmap of the Age-standardized Disability-Adjusted Life Years (DALYs) rate for malaria in males with respect to 13 variables.


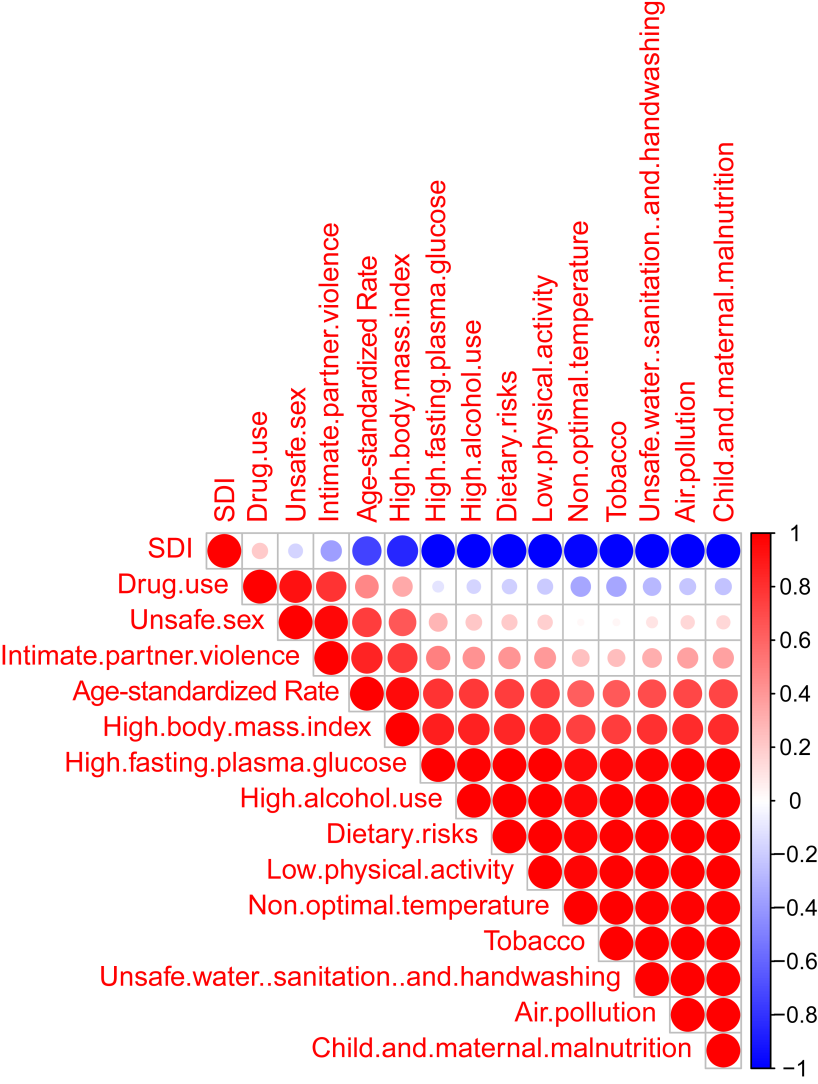


Fig.S2 The correlation heatmap of the Age-standardized Disability-Adjusted Life Years (DALYs) rate for malaria in females with respect to 13 variables.


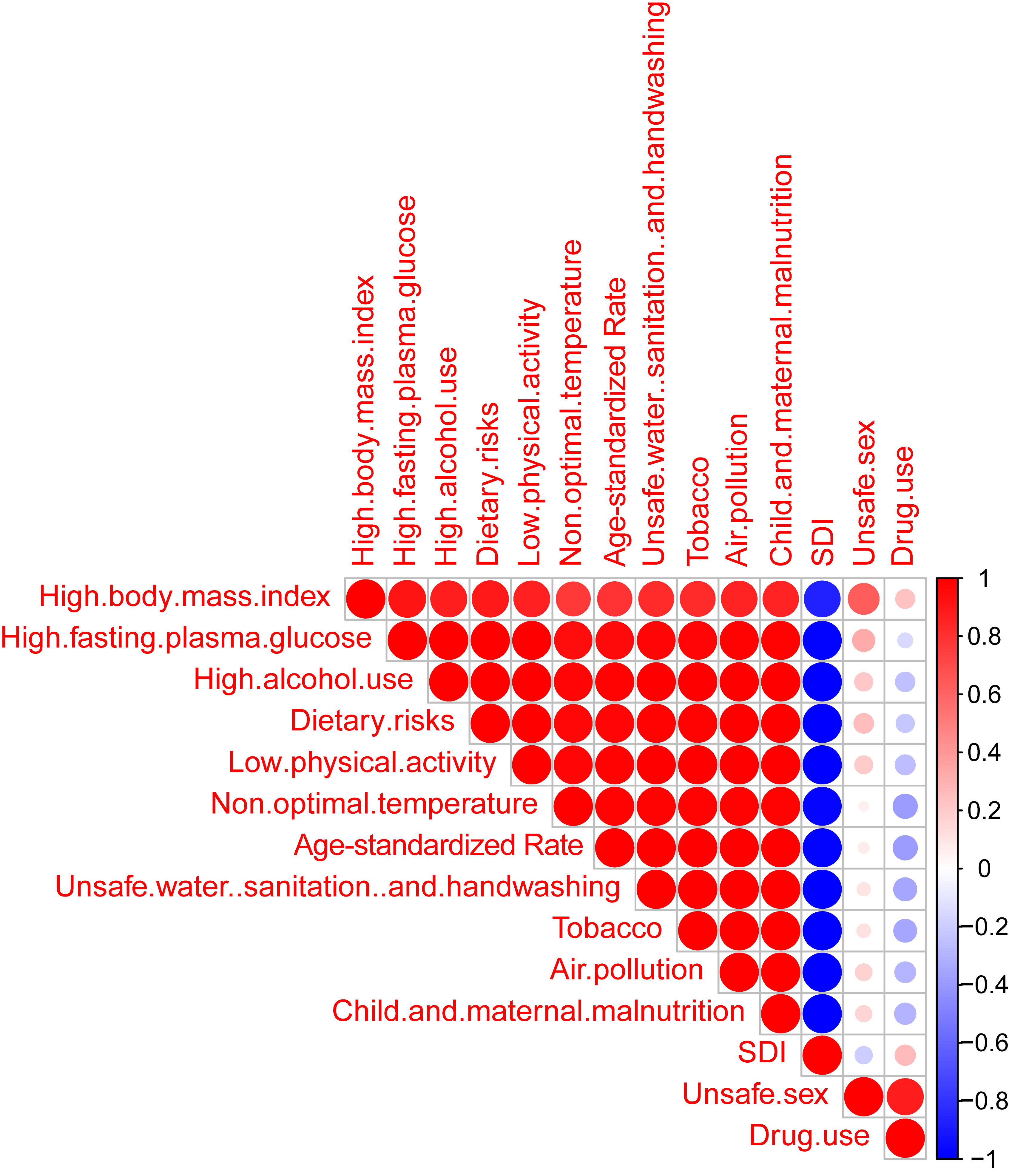


Fig.S3 The correlation heatmap of the Age-standardized Disability-Adjusted Life Years (DALYs) rate for NTDs in males with respect to 13 variables.


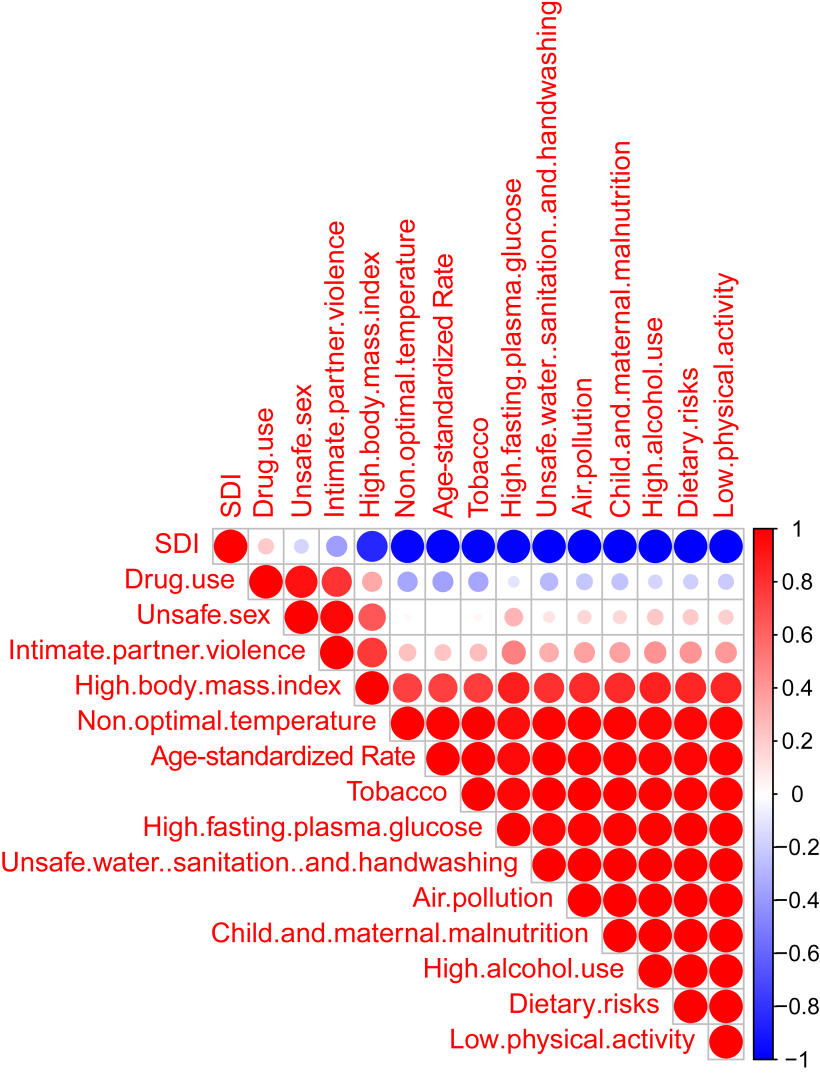


Fig.S4 The correlation heatmap of the Age-standardized Disability-Adjusted Life Years (DALYs) rate for NTDs in females with respect to 13 variables.

For a VAR(p) model, the formula is given by:

$$\text{y}_{\text{t}}=a_{0}+\sum_{j=1}^{p} y_{t-j}+u_{t}$$

where *y_t_* is a vector of endogenous variables at time *t*, a_0_ is a vector of constants, *A_j_* are coefficient matrices for the j-th lag, and *u_t_* is a vector of error terms at time *t*. The model can be rewritten using the lag operator *L* as:

$$\tilde{A}(L)y_{t}=a_{0}+u_{t}$$

where $\tilde{A}(L)=(I_{N}-A_{1}L-...-A_{P}L^{P})$. Assuming that *y_t_* is weakly stationary, the mean of *y_t_* can be expressed as:

$$\mu=E\left[\text{y}_{\text{t}}\right]=(I_{N}-A_{1}L-...A_{p}L^{p})^{-1}a_{0}$$

Defining$\tilde{\text{y}}_{\text{t}}=y_{t}-\mu$ , the model becomes:

The stationarity condition for the VAR(p) model requires that all roots of the characteristic equation det$\left(I_{N}-A_{1}\text{z}-...-A_{p}z^{p}\right)=0$lie outside the unit circle .{Lütkepohl, 2005 #52}^1-3^

The VAR model formula for NTDs and Malaria can be expressed as follows:

①The Age-standardized DALYs rate for Malaria：
②The Age-standardized DALYs rate for NTDs：

In this context, *y_1t_* represents the vector of age-standardized DALYs rate at time *t*, *y_2_* denotes the Socio-demographic Index (SDI), *y_3_* signifies unsafe water, sanitation, and hygiene practices, and *y_4_* indicates unsafe sexual behavior. The coefficient matrices β_i_ correspond to the lagged coefficients for the i-th lag, and ε*t* represents the vector of error terms at time *t*.

**Rolling forecast models** are a powerful tool for time series analysis, particularly when dealing with non-stationary data or when the relationships between variables change over time. The basic steps for implementing a rolling forecast model are as follows:

**1. Set Initial Training Set Size:** Choose an initial training set size (e.g., select the first T_0_ samples).

**2. Rolling Window:** After each prediction, add a new time point to the training set and discard the oldest data point, keeping the training set size constant.

**3. Model Training and Forecasting:** Train the model using the training set and make a forecast for the next one or more time steps.

**4. Error Calculation:** Calculate the error between the predicted values and the actual observed values.

**5. Repeat Steps:** Roll the window forward and repeat steps 2 to 4 until the entire forecasting process is completed.

Mathematically, let's assume:

- The time series data is where *t* is the time step.

- The initial training set size is *T_0_*, which is the number of time series data points used initially.

- The forecasting horizon is *h*, which is the number of future time steps to predict each time.

- The rolling window length is *T*, which is updated after each prediction.

- $\tilde{\text{y}}_{t+h}$is the predicted value for the future *h* time steps at time *t*.

The rolling forecast process can be summarized as:

1. Start with an initial training set {$y_{1},y_{2},...y_{T_{0}}$}.

2. Train the model on this initial set and predict .

3. Update the training set to{$y_{2},y_{3},...y_{T_{0}+1}$} and repeat the prediction step.

4. Continue this process, updating the training set and making predictions until the end of the time series is reached.

This approach allows for the assessment of the model's performance over time and its ability to adapt to new information, which is crucial for making informed decisions based on the most recent data.

The rolling forecast process can be expressed as follows:

1. Establish the Initial Training Set: $T\text{r}ain_{0}=\left\{y_{1}\right.,y_{2},...\left.y_{T_{0}}\right\}$.Use the initial training set to train the model and make predictions.

2. Initial Predictions:

3. Update the Training Set and Make Predictions: After each prediction, add a new observation to the training set, remove the oldest data point, and then make predictions based on the new training set.

4. First Update:$T\text{r}ain_{1}=\left\{y_{2}\right.,y_{3},...\left.y_{T_{0}+1}\right\}$.

Train a new model and predict future values:

5. Repeat the Process: Continue rolling the window forward and repeat steps 3 and 4 until the entire time series is forecasted:

Error Metrics:

During the rolling forecast process, we typically calculate the error for each round of predictions to assess the forecasting performance. Common error metrics include:

- Mean Squared Error (MSE):, where$\text{y}_{t}$is the actual value and$\hat{\text{y}}_{t}$is the predicted value.

- Root Mean Squared Error (RMSE):$RMSE=\sqrt{MSE}$

- Mean Absolute Error (MAE):

Example of Rolling Forecast Mathematical Formula:

Assume we have a time series dataset{$y_{1},y_{2},...y_{T}$} and the goal is to perform rolling forecasts.

1. Initial Training Set Size$T_{0}$: Choose the initial training set size, for example, $T_{0}=20$.

2. Forecasting Horizon$\text{h}$: Assume we predict data for the next 5 time steps $\left(\text{h}=5\right)$.

3. Rolling Window: Use$T_{0}$data points for each training model, then "roll" the window after each training.

**Process:**

- At time$\text{t}=T_{0}$, use {$y_{1},y_{2},...y_{T_{0}}$}to train the model and predict .

- At time $\text{t}=T_{0}+1$, update the training set to {$y_{2},y_{3},...y_{T_{0}+1}$} and predict .

- Continue this process until the model has rolled through all the data.

The advantage of rolling forecasts is that it not only provides predictions for the future but also adapts to changes in the time series data by continuously updating the model.

**Summary:**

The key idea of rolling forecasts is to dynamically update the training set and predict future values based on the latest data. It can effectively evaluate the model's predictive power in time series, especially when data points are limited. By updating the model with each roll, the model can adapt to changes in the data and continuously improve the forecasting effect.

1. Lütkepohl H. New Introduction to Multiple Time Series Analysis. 2005.

2. Kilian L, Lütkepohl H: Structural Vector Autoregressive Analysis. Cambridge: Cambridge University Press; 2017.

3. Chapter 3: Vector Autoregressive Methods [<https://phdinds-aim.github.io/time_series_handbook/03_VectorAutoregressiveModels/03_VectorAutoregressiveMethods.html#references>] （December28, data last accessed)
